# Anti-Trypanosomatid Elemanolide Sesquiterpene Lactones from *Vernonia lasiopus* O. Hoffm ^†^

**DOI:** 10.3390/molecules22040597

**Published:** 2017-04-08

**Authors:** Njogu M. Kimani, Josphat C. Matasyoh, Marcel Kaiser, Reto Brun, Thomas J. Schmidt

**Affiliations:** 1 Institute of Pharmaceutical Biology and Phytochemistry (IPBP), University of Münster, PharmaCampus Corrensstraße 48, D-48149 Münster, Germany; m_kima01@uni-muenster.de; 2 Department of Chemistry, Egerton University, P.O. Box 536, Egerton, Nairobi 20115, Kenya; josphat2001@yahoo.com; 3 Swiss Tropical and Public Health Institute (Swiss TPH), Socinstr. 57, CH-4051 Basel, Switzerland; marcel.kaiser@unibas.ch (M.K.); reto.brun@unibas.ch (R.B.); 4 University of Basel, Petersplatz 1, CH-4003 Basel, Switzerland

**Keywords:** *Vernonia lasiopus*, antiprotozoal activity, *Trypanosoma brucei rhodesiense*, *Trypanosoma cruzi*, *Leishmania donovani*, *Plasmodium falciparum*, elemanolide, sesquiterpene lactones

## Abstract

Sleeping sickness or human African trypanosomiasis (HAT) is a neglected tropical disease (NTD) threatening millions of peoples’ lives with thousands infected. The disease is endemic in poorly developed regions of sub-Saharan Africa and is caused by the kinetoplastid “protozoan” parasite *Trypanosoma brucei*. The parasites are transmitted to humans through bites of infected tsetse flies of the genus *Glossina*. The few available drugs for treatment of this disease are highly toxic, difficult to administer, costly and unavailable to poor rural communities bearing the major burden of this infection. Therefore, the search for new efficacious, safe and affordable drugs is of high importance. *Vernonia lasiopus* O. Hoffm., an indigenous African plant of the Asteraceae family, has been extensively reported to be used ethno-medicinally as a treatment for malaria. Its crude extracts obtained with solvents of different polarity were screened in vitro for anti-protozoal activity and the dichloromethane extract was found to be particularly active against *Trypanosoma brucei rhodesiense* (IC_50_ = 0.17 µg/mL). Bioassay-guided chromatographic fractionation of the dichloromethane extract led to the isolation and identification of six elemanolide type sesquiterpene lactones: 8-desacylvernolide, vernolepin, vernomenin, vernodalol, vernodalin and 11,13-dihydrovernodalin. All these elemanolide sesquiterpene lactones showed in vitro anti-trypanosomal activity. They were also tested for cytotoxicity against mammalian cells (L6 cell line). Vernolepin, the main component in the extract, was also the most potent with an IC_50_ value of 0.05 µg/mL against *T.b. rhodesiense* trypomastigotes. This compound showed a selectivity index of 14.5, which makes it an interesting candidate for in vivo tests and determination of its mechanism of action.

## 1. Introduction

Human African trypanosomiasis (HAT), also commonly known as sleeping sickness, is one of the neglected tropical diseases (NTDs) according to the World Health Organization (WHO) [1]. NTDs are ailments that afflict the poorest of the world’s communities which represent a non-lucrative sector for investment in drug development [2,3]. NTDs, besides being life-threatening infections, promote and advance poverty by disabling people, reducing their productivity in the case of adults and affecting learning, cognition and development in children, thus condemning these populations to more poverty [4].

HAT is caused by two trypanosomatid parasites, *Trypanosoma brucei rhodesiense* and *T. b. gambiense* in East and West Africa, respectively [5]. These “protozoans” are transmitted to humans by the bites of infected tsetse flies of the genus *Glossina* occurring in 36 sub-Saharan African countries. The disease mainly burdens poor rural communities who lack adequate access to health facilities. Through sustained efforts, the reported cases have been on the decline with only 3796 cases in 2014 [5]. However, it is believed that many cases go undiagnosed and unreported with an estimated population of about 70 million people at risk of infection [6]. For the treatment of HAT, only few drugs are available [2,3]. These drugs are very costly, often unavailable, highly toxic and difficult to administer, particularly in the second stage of infection. Furthermore, an increasing number of relapses are reported after treatment [2,3,5]. It is therefore clear that the search for leads towards new, affordable and available, safe and efficacious drugs is of high importance [3,7,8].

Natural products from plants, microorganisms and marine organism have a long and successful tradition as drugs or as leads in drug development [7,8]. In continuation of our ongoing search for new anti-trypanosomal agents in plants [9,10,11,12], we set out to investigate the activity of *Vernonia lasiopus* O. Hoffm. (Asteraceae) and its secondary metabolites against protozoan parasites. *V. lasiopus* is an African indigenous shrub that reaches up to 3 m in height [13]. It belongs to the Vernonieae tribe in the Asteraceae family [14]. The plant is used ethnomedicinally to cure indigestion, stomach-ache, gastrointestinal problems, worms, malaria, scabies, venereal diseases, sores and also as a purgative [13,15,16,17,18]. Organic crude extracts from this plant have been reported to possess antiplasmodial potency and cytotoxic activity in vitro [15,17,19,20]. No previous reports exist in the literature with respect to an anti-trypanosomal activity of *V. lasiopus* or its chemical constituents.

## 2. Results and Discussion

### 2.1. Antiprotozoal Activity of V. lasiopus Crude Extracts

Crude extracts of the aerial parts of *V. lasiopus* obtained with solvents of increasing polarity (n-hexane (Hex), dichloromethane (DCM), ethyl acetate (EtOAc), n-butanol (BuOH), methanol (MeOH) and water (H_2_O)) were tested for in vitro activity against *T. b. rhodesiense (Tbr)*, *T. cruzi (Tcr)*, *Leishmania donovani (Ldon)* and *Plasmodium falciparum (Pf)*. For these tests, *Tbr* (STIB 900 strain) trypomastigotes, *Tcr* (Tulahuen C4 strain) amastigotes, *Ldon* (MHOM-ET-67/L82 strain) amastigotes, *Pf* (NF54 strain) intra-erythrocytic forms, and for cytotoxicity L6 rat-skeletal myoblasts were used. The results are summarized in Table 1. It becomes evident that the DCM extract showed the highest activity against *Tbr* with an IC_50_ value of 0.17 µg/mL. It also displayed some lower activity against *Tcr* and *Pf*. For this reason, bioassay-guided fractionation, isolation and characterization of the potentially active constituents of the DCM extracts was undertaken.

### 2.2. Bioassay-Guided Fractionation and Isolation of Elemanolide Sesquiterpene Lactones

The DCM extract was fractionated into twelve major fractions by column chromatography on silica. Representative fractions were subjected to bioassay against *Tbr* (Table 2). The fractions were also analyzed by UHPLC/+ESI QTOF MSMS.

**Figure 1:**
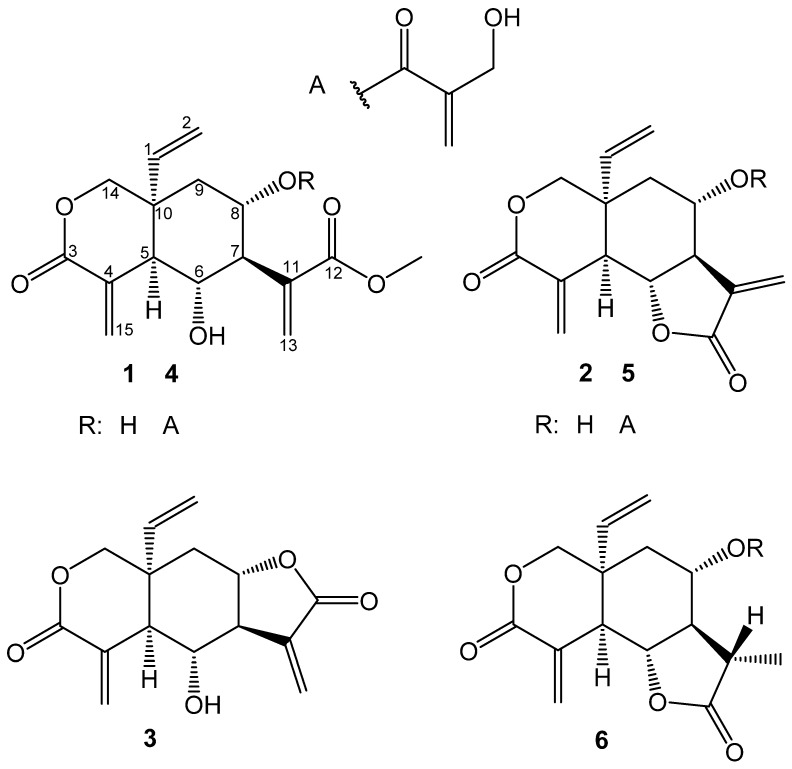
Elemanolide sesquiterpene lactones isolated from *V. lasiopus*.

The anti-trypanosomal activity was concentrated between fractions five and nine. Analysis of these fractions by UHPLC/+ESI QTOF MS/MS showed that all the major components in the crude extract were distributed among these fractions. Subsequently, these major components (compounds **1**–**6,** see Figure 1) were isolated. Compounds **5** and **6** were obtained only as an inseparable mixture in a ratio of approximately 2:1. The compounds **2**, **3**, **5**, and **6** were unambiguously identified as vernolepin (**2**), vernomenin (**3**), vernodalin (**5**) and 11,13-dihydrovernodalin (**6**), on the grounds of their 1D and 2D NMR data in conjunction with their exact masses as obtained from UHPLC/+ESI QTOF MS/MS analyses, which were in full agreement with literature values [21,22,23,24,25].

For compounds **1** and **4**, the proton NMR data were similar to those of two compounds with the same structure reported from *V. scorpioides* [22]. Incidentally, these data were also identical to their C-10 epimers, epivernodalol and lasiopulide, earlier reported in *V. lasiopus* [14]. To eliminate this ambiguity, where the two sets of epimers are reported to display the same chemical shifts and ^3^J_HH_ values of H-1 and H-2, a ^1^H/^1^H NOESY experiment for compound **1** was performed. From this, a correlation between H-1 and H-5 was noted, meaning that the two rings must be *cis*-fused. Therefore, by extension, the rings in compound **4**, in view of the very similar NMR signals, can be expected to be *cis*-fused as well. Therefore, these compounds were confidently confirmed to be 8-desacylvernolide (**1**) and vernodalol (**4**) and the earlier report [14] is probably to be revised. The other compounds, **2**, **3**, **5** and **6**, are all *cis*-fused as well, without ambiguity in the literature data [21,22,23,24,25].

### 2.3. Anti-Trypanosomal Activity of Isolated Elemanolides

All isolated compounds were tested for anti-trypanosomal activity and showed considerable degrees of activity. Vernolepin (**2**) was the most active with an IC_50_ value of 0.05 µg/mL (0.19 µM) and a selectivity index of 14.5. It is important to note that **2** was the major constituent of the DCM extract. This compound was approximately 3.5 times more active than the crude extract and it presents a ten-fold higher selectivity. It can therefore be concluded that vernolepin is a main contributor to the activity of extract. The other compounds showed slightly lower activity as well as lower selectivity. The full results are summarized in Table 3.

The mixture of vernodalin (**5**) and 11,13-Dihydrovernodalin (**6**) was the second most active with an IC_50_ value of 0.069 µg/mL. Vernodalin (**5**) has been previously reported to have anti-plasmodial activity with an IC_50_ value of 4.0 µg/mL against *P. falciparum* (multidrug resistance strain K1) [26]. The 11,13-Dihydrovernodalin (**6**) has also been reported to be active against *P. falciparum* chloroquine sensitive strain PoW and chloroquine-resistant strain Dd2 with IC_50_ values of 2.3 µg/mL and 1.1 µg/mL respectively [27].

Vernodalol (**4**) was the third most potent compound with an IC_50_ value of 0.1 µg/mL (0.26 µM). This compound has been reported to have in vitro anti-plasmodial activity against *P. falciparum* (multidrug resistance strain K1) with an IC_50_ value of 4.2 µg/mL [26]. Compound **1** was the least active with an IC_50_ value of 0.779 µg/mL (2.5 µM) against *Tbr.* Comparing the activity of compounds **1** and **4**, it is evident that compound **4** is more active by a factor of almost 10. This can be probably attributed to the ester moiety at C-8, which has an extra α, β-unsaturated carbonyl group. A similar trend could be expected for compounds **2** and **5**. However, compound **5** was only tested in the 2:1 mixture with **6** so that such a correlation cannot be made with certainty.

Vernolepin (**2**) was chosen for in vivo studies due to its high activity and considerable selectivity. Its mechanism of action will also be evaluated and studies in this direction are in progress.

## 3. Materials and Methods

### 3.1. General Experimental Procedures

Analytical TLC was performed on silica gel plates 60 F254 (Merck Chemicals GmbH, Darmstadt, Germany) with various solvent systems consisting of Ethylacetate and hexane as the mobile phase. The plates were visualized under UV-light at 254/360 nm and then sprayed with anisaldehyde/sulfuric acid reagent and heated on a hot plate.

### 3.2. Plant Material

The aerial parts of the *V. lasiopus* O. Hoffm. plants were collected from Egerton University botanic garden in Njoro Kenya (0°21′44.8″ S 35°55′26.9″ E). The plants were identified by a taxonomist at the Biological Sciences Department at Egerton University. They were then air dried under shade at ambient temperature to constant weight. The dried materials were then powdered with a mill to 1 mm mesh size.

### 3.3. Extraction and Isolation

#### 3.3.1. Small-Scale Extraction for Initial Bioassays

For the initial bioassays, the ground air-dried materials (100 g) were macerated sequentially with solvents (350 mL each) of increasing polarity (hexane, dichloromethane (DCM), ethyl acetate and methanol) for 48 h at room temperature with intermittent shaking. The extracts were filtered over whatman no. 1 filter paper, the solvent was removed under reduced pressure at 40 °C and the extracts were dried further in vacuo. The extracts were then tested for in vitro activity against *T. b. rhodesiense, T. cruzi, L. donovani* and *P. falciparum* (see Table 1). The DCM extract showed high potency against *T. b. rhodesiense* and was thus selected for bioassay-guided fractionation and isolation at a larger scale.

#### 3.3.2. Soxhlet Extraction for Preparative Isolation

*V. lasiopus* dry powdered plant material (750 g, in portions of 250 g) was exhaustively extracted in a Soxhlet-apparatus for approximately 13 h with dichloromethane (total 5 L). The solvent was evaporated in vacuo at 40 °C to 76.45 g (10.1%) of extract. The extract (20 g) was fractionated by column chromatography on silica gel 60 (1250 g). The column was eluted with solvents of increasing polarity (100% hexane−100% ethyl acetate) of hexane–EtOAc mixture (*v*/*v*) as follows: 3 L hexane, 5 L (7:3), 2.5 L (1:1), 2.5 L (3:7), and 2.5 L EtOAc. The flow was maintained at about 1 mL/min. After collection of about 2.5 L of pre-eluate, 320 eluates of around 30 mL were collected and pooled into twelve fractions (F1–F12) according to their TLC profiles. Representative fractions were tested for anti-trypanosomal activity (see Table 2). After LC-Ms analysis of fractions F1–F12, fractions F6–F9 were recombined (4.8 g) and re-chromatographed over 250 g silica gel 60. The column was eluted with 4.5 L hexane–EtOAC–methanol mixture (7:4:1) at a flow rate of about 1 mL/min. A hundred and forty eluates of about 30 mL were collected. Compound **2** crystallized from eluates 45–65. The crystals were washed in hexane and re-crystallized in hexane: Ethyl acetate mixture (7:3). According to the TLC profiles of the rest of the eluates, they were combined in three sub-fractions F7a (980 mg), F7b (1.1 g) and F7c (995.9 mg). Sub fraction F7b was further separated on preparative HPLC to yield compounds **1** (6.2 mg) and **2** (38.6 mg). Sub fraction F7a was also separated by preparative HPLC to obtain compounds **3** (5.2 mg), **4** (5.6 mg) and a mixture of **5** and **6** (23.4 mg).

#### 3.3.3. Purification by Preparative High Performance Liquid Chromatography (HPLC/UV-DAD)

The preparative HPLC isolation was performed on a Jasco (Groß-Umstadt, Germany) preparative HPLC system (pump: PU-2087 plus; diode array detector MD 2018 plus; column thermostat CO 2060 plus; autosampler AS 2055 plus; LC Net II ADC Chromatography Data Solutions; sample injection loop: 2000 µL) on a preparative reverse phase column Reprosil 100 C-18 (5 µm, 250 mm × 20 mm, Macherey-Nagel, Düren, Germany) with binary gradients of the mobile phase. The optimized mobile phase was composed of water (A) and methanol (B) using the following gradient conditions: 50%–55% of B (0 to 10 min) which was held for five minutes, 55%–65% of B (15 to 20 min), 65%–80% of B (20–25 min), 80%–100% of B (25 to 30 min), 100% of B (30 to 34 min) and another four minutes to return to the initial conditions. A flow rate of 9 mL/min and a column temperature of 40 °C were used in all separations. Chromatograms were recorded at 220, 254 and 265 nm. The retention times of compounds **1**–**6** were 8.42 min (**1**), 9.75 min (**2**), 10.82 min (**3**), 12.92 min (**4**), and 14.96 min (**5** + **6**), respectively.

### 3.4. Analysis of the Plant Extracts and Isolated Compound by UHPLC/+ESI-QTOF-MS/MS

The DCM extract, collected fractions and isolated compounds were dissolved in methanol at concentrations of 10 mg/mL and 0.1 mg/mL for crude extract or fractions, and for pure compounds, respectively. Chromatographic separations were performed on a Dionex Ultimate 3000 RS Liquid Chromatography System (Idstein, Germany) with a Dionex Acclaim RSLC 120, C18 column (2.1 × 100 mm, 2.2 µm) using a binary gradient (A: water with 0.1% formic acid; B: acetonitrile with 0.1% formic acid) at 0.8 mL/min: 0–9.5 min: linear from 5% B–100% B; 9.5–12.5 min: isocratic 100% B; 12.5–12.6 min: linear from 100% B down to 5% B; 12.6–15 min: isocratic 5% B. The injection volume was 5 µL. Eluted compounds were detected using a Dionex Ultimate DAD-3000 RS over a wavelength range of 200–400 nm and a Bruker Daltonics micrOTOF-QII quadrupole/time-of-flight mass spectrometer (Bremen, Germany) equipped with an Apollo electrospray ionization source in positive mode at 5 Hz over a mass range of *m*/*z* 50–1000 using the following instrument settings: nebulizer gas nitrogen, 5 bar; dry gas nitrogen, 9 L/min, 220 °C; capillary voltage 4500 V; end plate offset −500 V; transfer time 70 µs; collision gas nitrogen; collision energy and collision radio frequency (RF) settings were combined for each single spectrum of 1000 summations as follows: 250 summations with 20% base collision energy; 130 Vpp+ 250 summations with 100% base collision energy; 500 Vpp+ 250 summations with 20% base collision energy; and 130 Vpp + 250 summations with 100% base collision energy and 500 Vpp. Base collision energy was 50 eV for precursor ions with a *m*/*z* less than 500 and then linearly interpolated against *m*/*z* up to a maximum of 70 eV for precursor ions with a *m*/*z* of up to 1000. Internal dataset calibration (HPCmode) was performed for each analysis using the mass spectrum of a 10 mM solution of sodium formate in 50% isopropanol that was infused during LC re-equilibration using a divert valve equipped with a 20-µL sample loop. The retention times of compounds **1**–**6** are reported below (also see Supplementary materials).

### 3.5. Structural Analysis of Isolated Compounds

NMR spectra (^1^H, ^13^C, ^1^H/^1^H COSY, ^1^H/^1^H NOESY, ^1^H/^13^C HSQC, and ^1^H/^13^C HMBC) were recorded on a 600 MHz Agilent DD2 spectrometer. The spectra were obtained at 298 K in CDCl_3_. The CDCl_3_ solvent signals (^1^H; 7.260 ppm and ^13^C; 77.000 ppm) were used to reference the spectra. MestReNOVA v. 11 (Mestrelab Research, Chemistry Software Solutions, Santiago de Compostela, Spain) software was used to process and evaluate the spectra.

### 3.6. Analytical Data

*8-Desacylvernodalol* **1** UHPLC/+ESI-QTOF MS: Rt 2.16 min, MS (*m*/*z*): 309.1356 [M + H]^+^; calcd. for C_16_H_21_O_6_^+^: 309.1338); ^1^H-NMR (600 MHz, CDCl_3_; δ (ppm), intensity, mult., *J* (Hz)): 6.57 (1H, d, 1.7, H-15a); 6.40 (1H, d, 1.0, H-13a); 5.79 (1H, d, 1.0 Hz, H-13b); 5.71 (1H, br. dd, 1.7, 1.0, H-15b); 5.68 (1H, ddd, 17.5, 10.9, 0.9, H-1); 5.21 (1H, dd, 17.5, 0.9, H-2a); 5.18 (1H, dd, 10.9, 0.9, H-2b); 4.54 (1H, dd, 11.8, 0.9, H-14β); 4.26 (1H, dd, 11.9, 2.1, H-14α); 4.05 (1H, m, H-8); 3.97 (1H, td, 10.5, 2.5, H-6); 3.74 (3H, s, H-12-OMe); 2.42 (2H, m; H-5,H-7); 1.93 (1H, dd, 14.0, 4.7, H-9β); 1.51 (1H, dd, 14.0, 11.5, H-9α).

*Vernolepin* **2** UHPLC/+ESI-QTOF MS: Rt 3.65 min, MS (*m*/*z*): 277.1111 [M + H]^+^; calcd. for C_15_H_17_O_5_^+^: 277.1076); ^1^H-NMR (600 MHz, CDCl_3_; δ (ppm), intensity, mult., *J* (Hz)): 6.75 (1H, d, 0.9, H-15a); 6.25 (1H, d, 3.2, H-13a); 6.03 (1H, d, 3.0, H-13b); 5.96 (1H, d, 1.1, H-15b); 5.75 (1H, dd, 17.5, 11.0, H-1); 5.30 (1H,d, 11.0, H-2a); 5.27 (1H, d, 17.4, H-2b); 4.40 (1H, d, 12.1, H-14β); 4.23 (1H, dd, 1.8, 12.2, H-14α); 4.08 (1H, tdd, 10.3, 5.5, 4.7, H-8); 3.93 (1H, t, 11.3, H-6); 2.98 (1H, dd, 11.3,1.9, H-5); 2.68 (1H, tt, *J* = 11.1, 10.3, 3.1, H-7); 1.99 (1H, dd, 14.2, 4.7, H-9β); 1.81 (1H, d, 5.5, H-8-OH);1.68 (1H, dd, 14.2, 10.4, H-9α).

*Vernomenin* **3** UHPLC/+ESI-QTOF MS: Rt 3.91 min, MS (*m*/*z*): 277.1093 [M + H]^+^; calcd. for C_15_H_17_O_5_^+^: 277.1076); ^1^H-NMR (600 MHz, CDCl_3_; δ (ppm), intensity, mult., *J* (Hz)): 6.70 (1H, dd, 1.6, 0.5, H-15a); 6.67 (1H, ddt, 11.4, 10.3, 3.0, H-7); 6.23 (1H, dd, 3.1, 0.7, H-13a); 6.04 (1H, dd, 3.0, 0.7, H-13b); 5.81 (1H, dd, 1.6, 0.9, H-15b); 5.73 (1H, ddd, 17.4, 10.9, 0.8, H-1); 5.29 (1H, d, 10.9, H-2a); 5.25 (1H, dd, 17.4, 0.6 Hz, H-2b); 4.44 (1H, dd, 12.0, 0.9, H-14β); 4.30 (1H, dd, 12.0, 2.0, H-14α); 4.00 (1H, ddd, 12.4, 11.3, 4.1, H-8); 3.90 (1H, ddd, 9.5,2.2, H-6); 2.59 (1H, dd, 1H, 9.5,2.2, H-5); 2.44 (1H, d, 3.7, H-6-OH); 2.17 (1H, dd, 13.2,4.1, H-9β); 1.89 (1H, t, 12.8, H-9α).

*Vernodalol* **4** UHPLC/+ESI-QTOF MS: Rt 4.63 min, MS (*m*/*z*): 393.1571 [M + H]^+^; calcd. for C_20_H_25_O_8_^+^: 393.1549); ^1^H-NMR (600 MHz, CDCl_3_; δ (ppm), intensity, mult., *J* (Hz)): 6.64 (1H, d, 0.6, H-15a); 6.33 (1H, d, 0.9, H-13a); 6.17 (1H, q, 1.1, H-4′a); 5.81 (1H, q, 1.4, H-4′b); 5.74 (2H, m, H-13b,15b); 5.68 (1H, ddd, 17.6, 11.0, 0.9, H-1); 5.35 (1H, td, 11.4, 4.9, H-8); 5.26 (1H, d, 17.5, H-2a); 5.23 (1H, d, 10.9, H-2b); 4.67 (1H, dd, 12.0, 1.0, H-14β ); 4.35 (1H, dd, 12.0, 2.2, H-14α); 4.26 (2H, s, H-3’); 4.11 (1H, q, 7.1, H-6); 3.76 (3H, s, 12-OMe); 2.73 (1H, t, 10.8, H-7); 2.50 (1H, dd, 10.2, 2.1 Hz, H-5); 2.07 (1H, dd, 13.8, 4.9, H-9β); 1.61 (1H, dd, 13.8, 2.8, H-9α).

*Vernodalin* **5** UHPLC/+ESI-QTOF MS: Rt 4.99 min, MS (*m*/*z*): 361.1324 [M + H]^+^; calcd. for C_19_H_21_O_7_^+^: 361.1287); ^1^H-NMR (600 MHz, CDCl_3_; δ (ppm), intensity, mult., *J* (Hz)): 6.70 (1H, t, 0.9, H-15a); 6.26 (1H, d, *J* = 1.1, H-4’a); 6.16 (1H, d, 3.2, H-13a); 5.94 (1H, d, 1.4, H-4′b); 5.91 (1H, t, 1.2, H-15b); 5.68 (1H, m, H-1); 5.62 (1H, d, 2.9, H-13b); 5.26 (2H, m, H-2); 5.15 (1H, dt, 10.5, 5.3, H-8); 4.48 (1H, d, 12.3, H-14β); 4.32 (2H, s, H-3’ ); 4.24 (1H, m, H-14α); 4.06 (1H, m, H-6); 3.01 (1H, dd, 11.2, 1.5, H-5); 2.97 (1H, m, H-7); 2.19 (1H, dd, 14.2,4.7, H-9β); 1.64 (1H, m, H-9α).

*11,13-Dihydrovernodalin* **6** UHPLC/+ESI-QTOF MS: Rt 5.04 min, MS (*m*/*z*): 363.1481[M + H]^+^; calcd. for C_19_H_23_O_7_^+^: 363.1444); ^1^H-NMR (600 MHz, CDCl_3_; δ (ppm), intensity, mult., *J* (Hz)): 6.68 (1H, t, 0.9, H-15a); 6.23 (1H, d, 1.1, H-4’a); 5.92 (1H, br. d, 1.0, H-4′b); 5.88 (1H, br. d, 1.3, H-15b); 5.69 (1H, m, H-1); 5.25 (2H, m, H-2); 5.09 (1H, td, 10.9, 4.6, H-8); 4.51 (1H, d, 12.2, H-14β); 4.29 (2H, s, H-3’); 4.24 (1H, m, H-14α); 4.10 (1H, m, H-6); 2.86 (1H, d, 11.2, H-5); 2.06 (1H, dd, 14.2, 4.6, H-7); 2.64 (1H, dq, 12.0,6.9, H-11); 2.19 (1H, dd, 14.2,4.7, H-9β); 1.64 (1H, m, H-9α); 1.22 (3H, d, 0.6, H-13).

All spectral data were in full agreement with literature data [14,21,22,23,24,25].

### 3.7. In Vitro Bioassays

In vitro assays for the bioactivity of crude extracts and elemanolide sesquiterpene lactones against *Trypanosoma brucei rhodesiense* (bloodstream trypomastigotes, STIB 900 strain), *T. cruzi* (amastigotes, Tulahuen C4 strain), *Leishmania donovani* (amastigotes, MHOM-ET-67/L82 strain), *Plasmodium falciparum* (intra erythrocytic forms, NF54 IEF strain), and cytotoxicity test against mammalian cells (L6-cell-line from rat-skeletal myoblasts) were performed at the Swiss Tropical and Public Health Institute (Swiss TPH, Basel, Switzerland) according to established protocols as described earlier [28].

## 5. Conclusions

The bioassay-guided fractionation of the dichloromethane extract of *V. lasiopus* leaves led to the isolation and characterization of six elemanolide sesquiterpene lactones (**1**–**6**). Compounds **5** and **6** were obtained as an inseparable mixture. Vernolepin (**2**) was the main constituent of the DCM extract and incidentally the most potent against *Tbr* with an in vitro IC_50_ value of 0.19 µM and a SI of 14.5. The 8-Desacylvernodalol **1** and vernodalol **4** also showed considerable in vitro activity against *Tbr* with SI values ˃10. Vernolepin (**2**) is an interesting candidate for in vivo and mechanism of action studies. These studies are already in progress. The bioactivity data obtained in this study complements previous data on sesquiterpene lactones obtained in our lab. Elemanolide type sesquiterpene lactones have not previously been reported to have anti-trypanosomal activity, therefore that the data reported here also broaden the knowledge on structure–anti-trypanosomal activity relationships of sesquiterpene lactones [10,28].

## Figures and Tables

**Table 1:** In vitro antiprotozoal and cytotoxic activity (IC_50_ values in µg/mL) of crude extracts from *V. lasiopus*. Selectivity indices (SI) represent the ratio of cytotoxic over antiprotozoal IC_50_ values. Data are means of two independent determinations ± standard deviation.

Extract	*Tbr*	*Tcr*	*Ldon*	*Pf*	Cytotox. L6
**Hex**	5.57 ± 0.750	55.0 ^a^	5.91 ^a^	11.8 ^a^	nd
**DCM**	0.170 ± 0.100	2.18 ^a^	1.88 ± 1.07	1.09 ^a^	0.800 ± 0.340
**EtOAC**	1.30 ± 0.830	17.4 ^a^	11.9 ^a^	7.01 ^a^	4.79 ± 0.260
**BuOH**	2.81 ± 1.29	27.4 ^a^	14.9 ^a^	14.4 ^a^	nd
**MeOH**	9.25 ± 3.27	65.8 ^a^	41.2 ^a^	27.0 ^a^	nd
**H_2_O**	46.3 ± 6.80	75.5 ^a^	>100 ^a^	>50 ^a^	nd
**Pos. Control**	0.003 ± 0.001	0.382 ^a^	0.091 ± 0.070	0.002 ± 0.000	0.007 ± 0.001

^a^ Only one replicate was used to determine IC_50_; nd: not determined; Positive controls: melarsoprol (*Tbr*), benznidazole (*Tcr*), miltefosine (*Ldon*), chloroquine (*Pf*), podophyllotoxin (Cytotox L6).

**Table 2:** In vitro and cytotoxic activity (IC_50_ values in µg/mL) of selected fractions of the DCM extract from *V. lasiopus.* For positive controls, see Table 1.

Fraction	*Tbr*	Cytotox. L6
**F1**	30.1 ^a^	nd
**F3**	14.2 ^a^	nd
**F5**	0.673 ± 0.170	2.90 ^a^
**F7**	0.523 ± 0.400	1.16 ± 0.590
**F8**	0.414 ± 0.200	0.769 ^a^
**F9**	0.312 ± 0.240	2.84 ± 1.35
**F11**	14.1 ^a^	nd
**F12**	4.37 ^a^	nd

^a^ Only one replicate was used to determine IC_50_; nd: not tested.

**Table 3:** In vitro anti-trypanosomal and cytotoxic IC_50_ values in µg/mL (values in µM of pure compounds are reported in brackets) of elemanolides **1**–**6**. For positive controls, see Table 1.

Compound	*Tbr*	Cytotox. L6	SI
**1**	0.779 ± 0.220 (2.53)	10.7 ± 2.05 (34.6)	13.7
**2**	0.051 ± 0.035 (0.185)	0.735 ± 0.052 (2.68)	14.5
**3**	0.140 ± 0.100 (0.508)	0.620 ± 0.013 (2.26)	4.50
**4**	0.100 ± 0.041 (0.255)	1.44 ± 0.099 (3.67)	14.4
**5** + **6** (2:1)	0.069 ± 0.005	0.532 ± 0.029	7.70

## Supplementary Materials

The NMR and mass spectra of all isolates are provided as supplementary Figures S1–S32 available online.
