# Supplementary material for: Anti-Trypanosomatid Elemanolide Sesquiterpene Lactones from Vernonia lasiopus O. Hoffm †
Source: Molecules. 2017 Apr 8;22(4):597. doi: 10.3390/molecules22040597 (PMC6154322; doi:10.3390/molecules22040597)
Supplement: Supplementary file 1 [file molecules-22-00597-s001.pdf]

Supplementary Material

Anti-trypanosomatid elemanolide sesquiterpene lactones from *Vernonia lasiopus* O. Hoffm.

Njogu M. Kimani[1], Josphat C. Matasyoh[2], Marcel Kaiser[3,4] Reto Brun[3,4] and Thomas J. Schmidt[1,*]

[1] Institute of Pharmaceutical Biology and Phytochemistry (IPBP), University of Münster, PharmaCampus Corrensstraße 48, Münster D-48149, Germany; Email: m_kima01@uni-muenster.de
[2] Department of Chemistry, Egerton University, P.O. Box 536, Egerton 20115, Kenya; Email: josphat2001@yahoo.com
[3] Swiss Tropical and Public Health Institute (Swiss TPH), Socinstr. 57, Basel CH-4051, Switzerland; Email: marcel.kaiser@unibas.ch (M.K.); reto.brun@unibas.ch (R.B.)
[4] University of Basel, Petersplatz 1, Basel CH-4003, Switzerland
* Correspondence: thomschm@uni-muenster.de; Tel.: +49-251-83-33378

Compound **1**

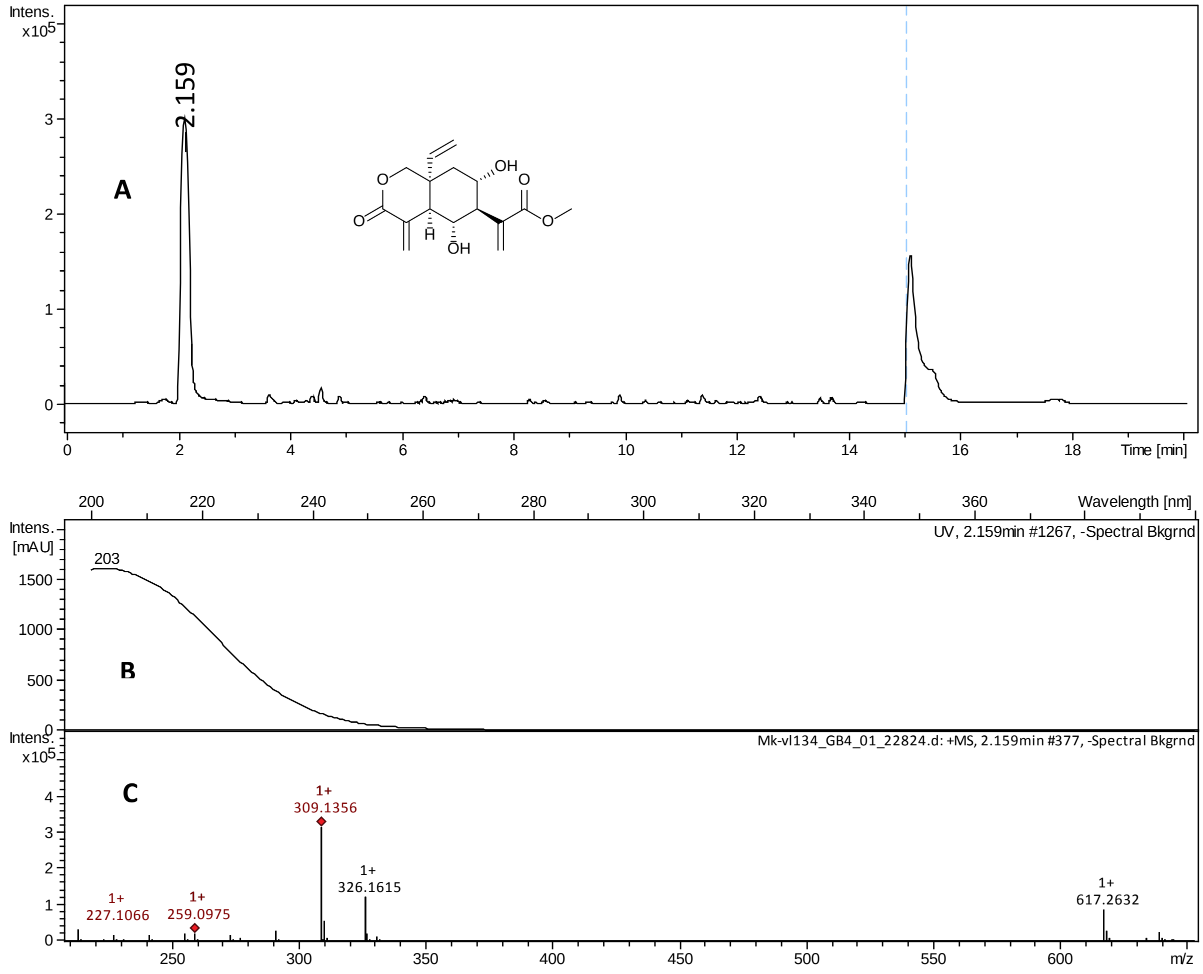

Figure S1: +ESI-QqTOF-MS chromatogram (**A**); UV spectrum (**B**) and +ESI-MS spectrum (**C**) of compound **1**, [M + H]+: 309.1356 ($C_{16}H_{21}O_6$). Internal calibrant sodium formate: 15.106 min

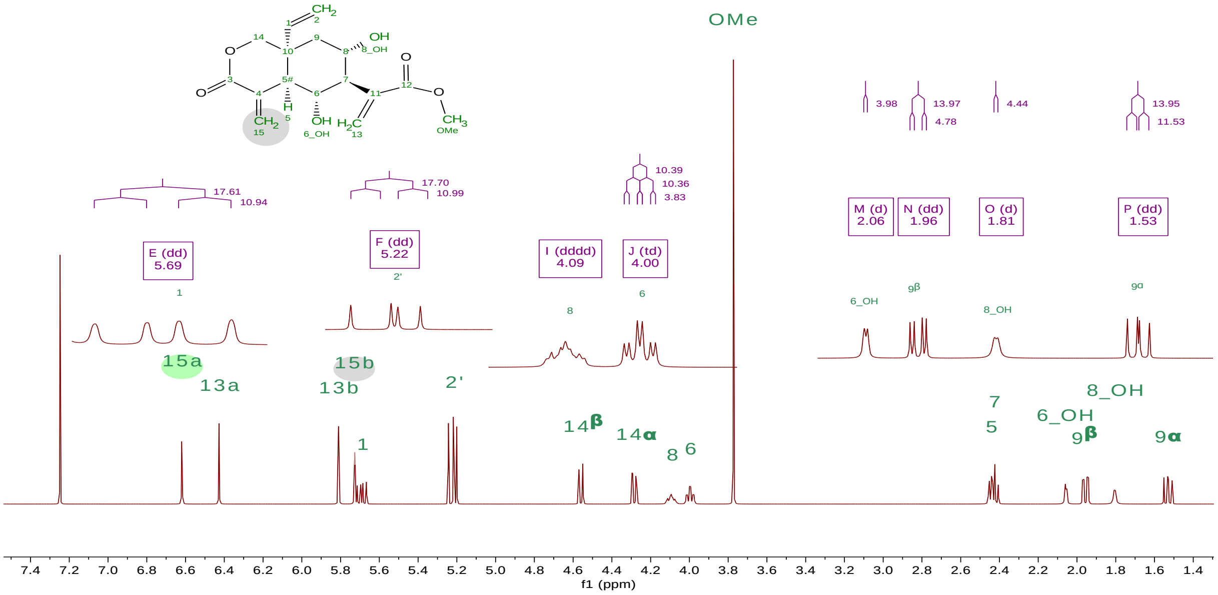

Figure S2: ^{1}H-NMR spectrum of compound **1** ($CDCl_3$, 600 MHz)

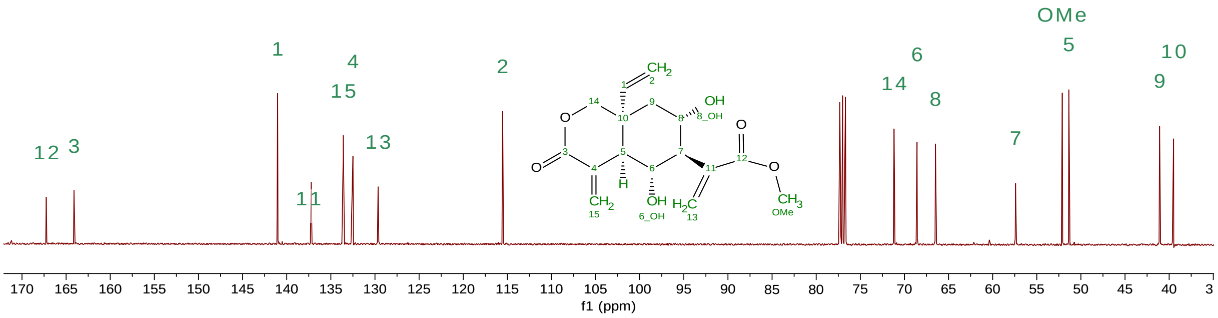

Figure 3: ^{13}C-NMR spectrum of compound **1** ($CDCl_3$, 150 MHz)

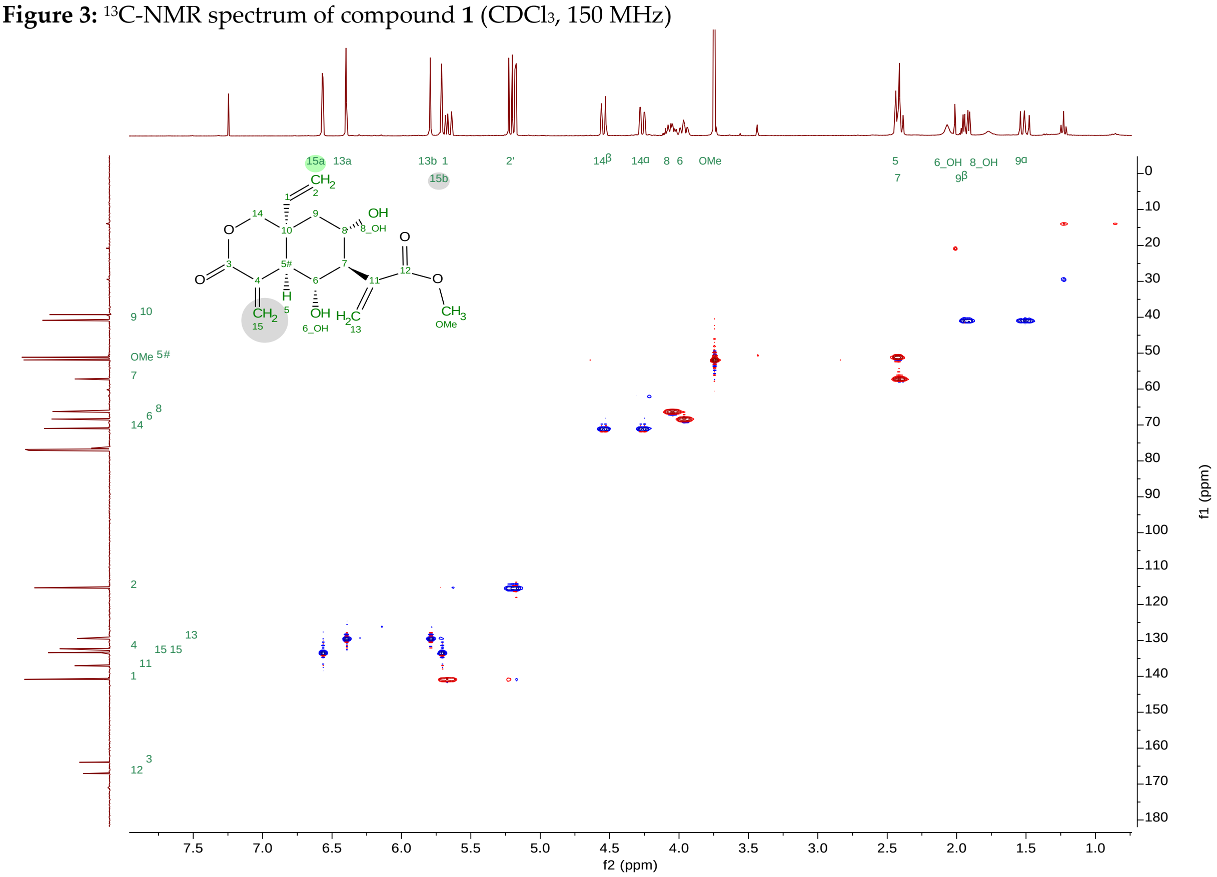

Figure S4: $^{1}H/^{13}C$-HSQC spectrum of compound **1** ($CDCl_3$, 600MHz)

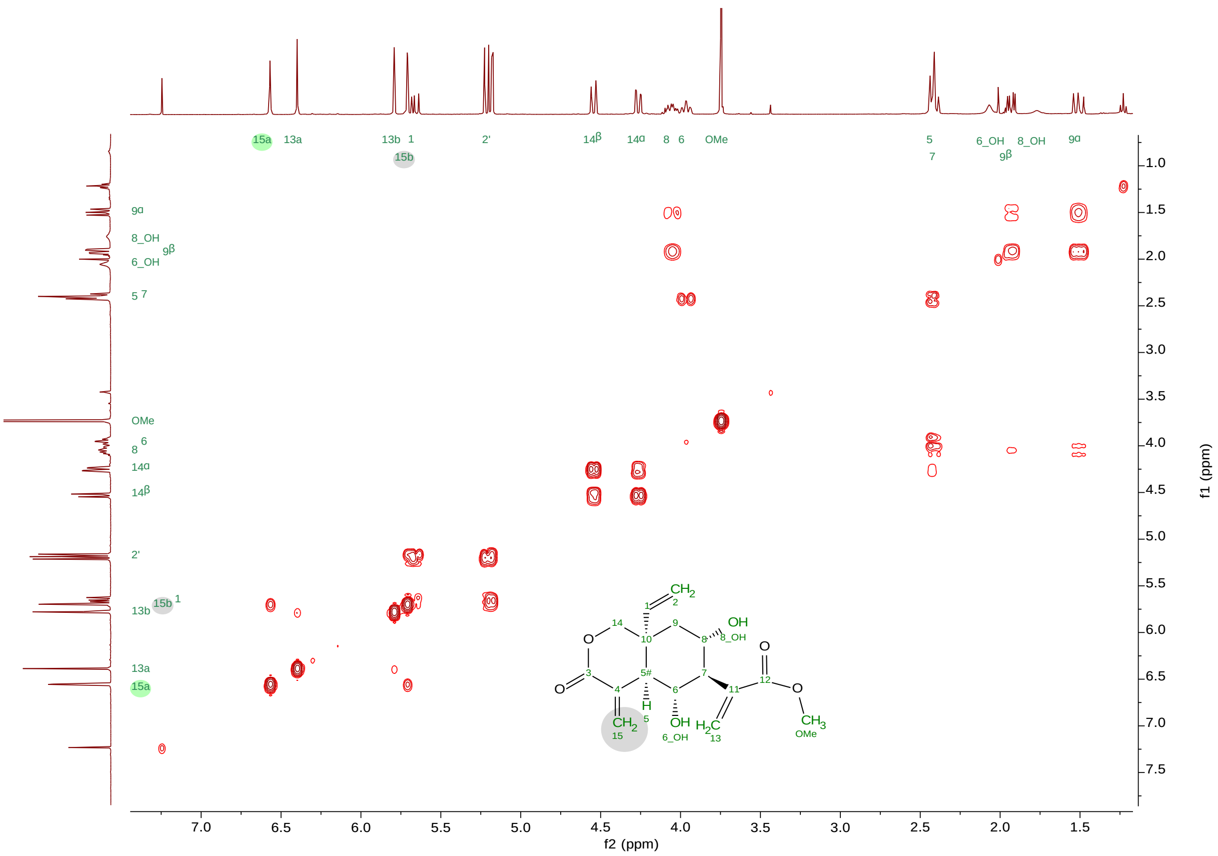

Figure S5: $^{1}H/^{1}H$- COSY spectrum of compound **1** ($CDCl_3$, 600MHz)

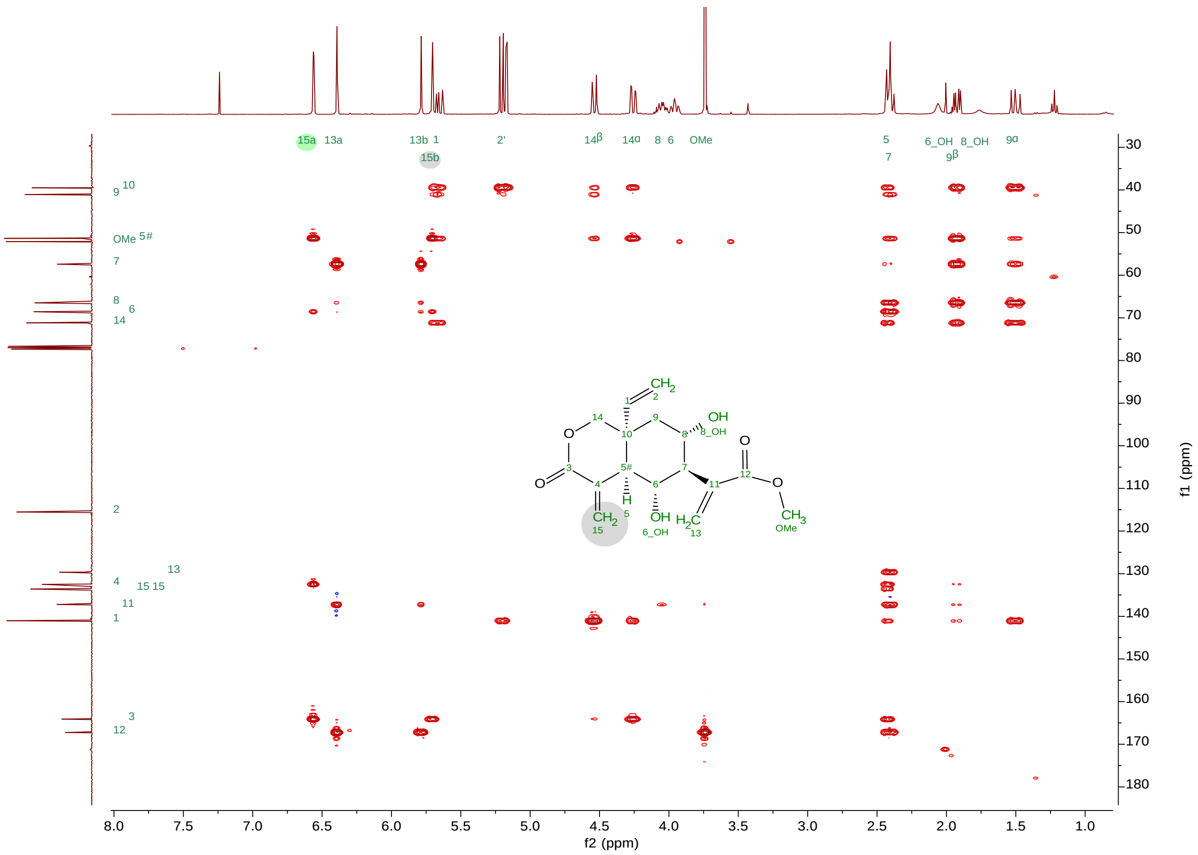

Figure S6: $^{1}H/^{13}C$- HMBC spectrum of compound **1** ($CDCl_3$, 600MHz)

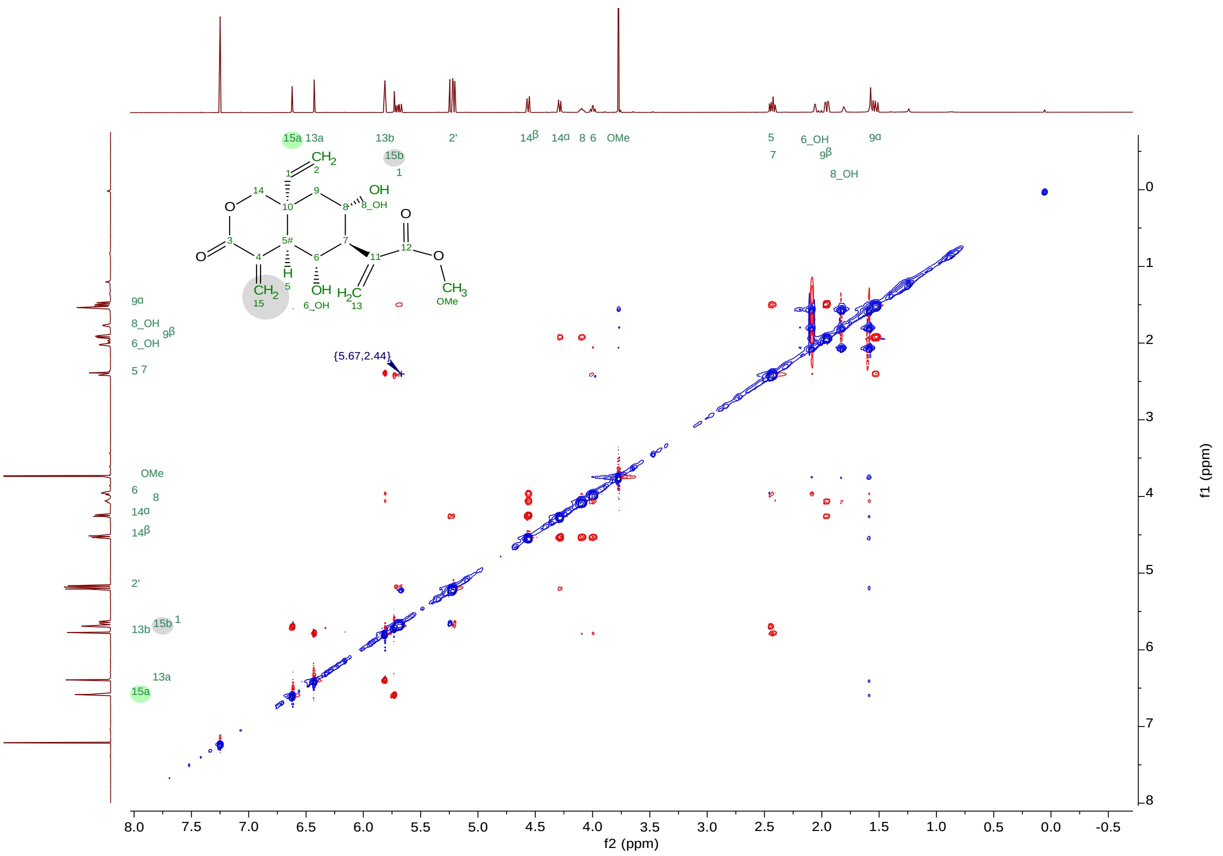

Figure S7: $^{1}H/^{1}H$- NOESY spectrum of compound **1** ($CDCl_3$, 600MHz)

Compound **2**

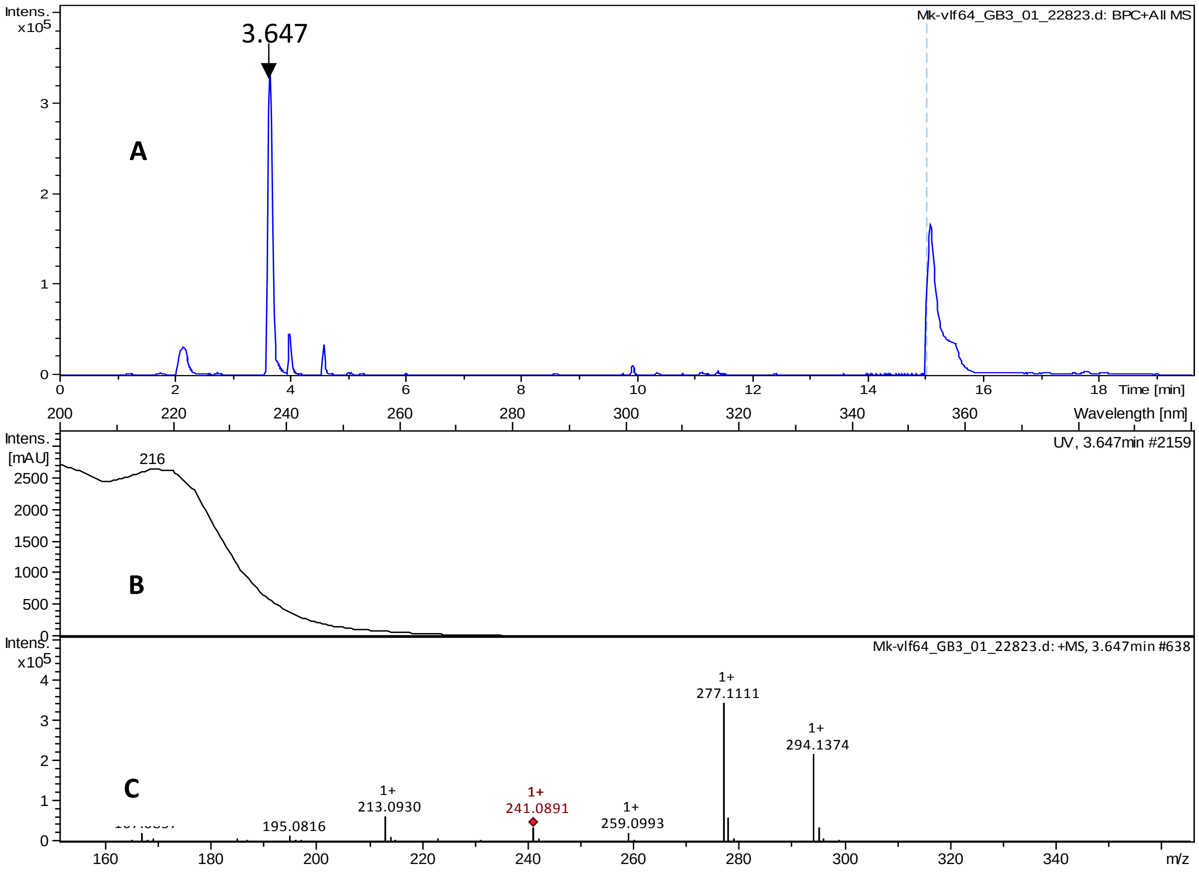

Figure S8: +ESI-QqTOF-MS chromatogram (**A**); UV spectrum (**B**) and +ESI-MS spectrum (**C**) of compound **2**, $[M + H]^+$: 277.1111 ($C_{15}H_{17}O_5$). Internal calibrant sodium formate: 15.106 min

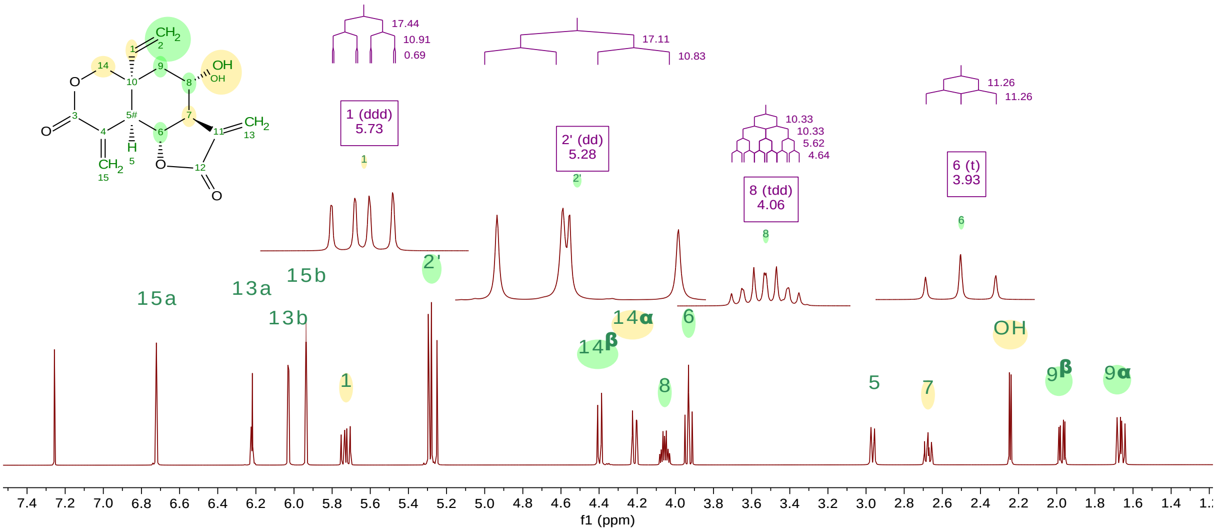

Figure S9: ^{1}H-NMR spectrum of compound **2** ($CDCl_3$, 600 MHz); *

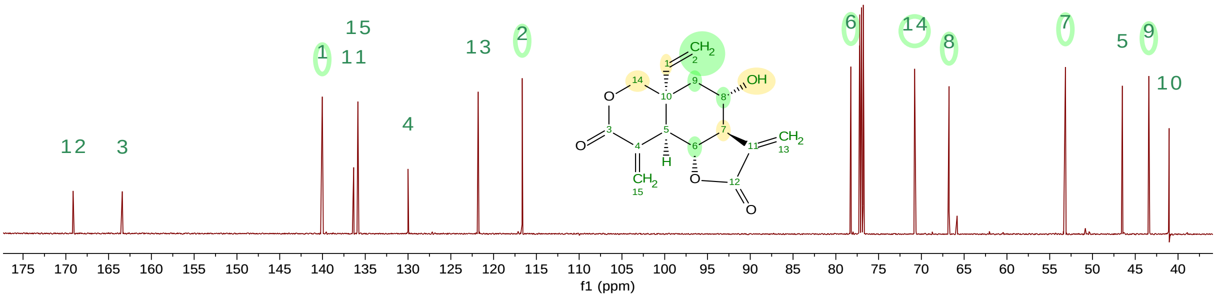

Figure S10: ^{13}C-NMR spectrum of compound **2** ($CDCl_3$, 600 MHz)

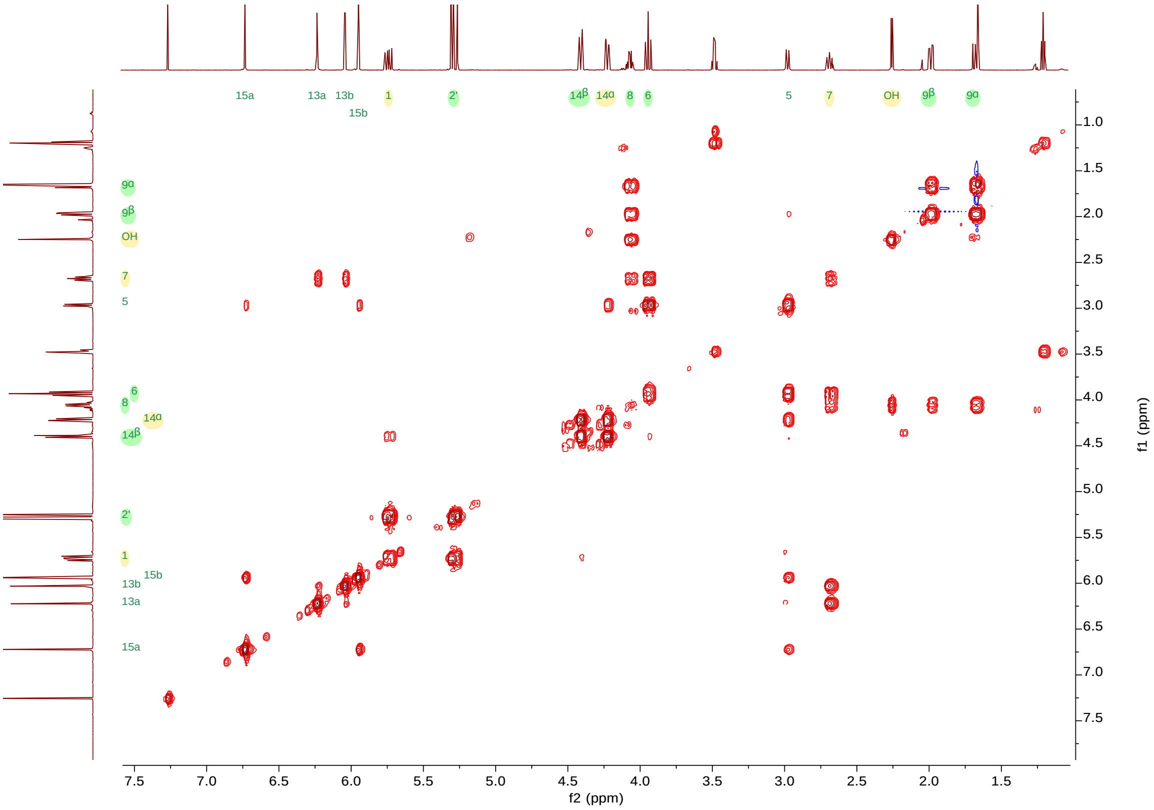

Figure S11: ^{1}H/^{1}H- COSY spectrum of compound **2** ($CDCl_3$, 600MHz)

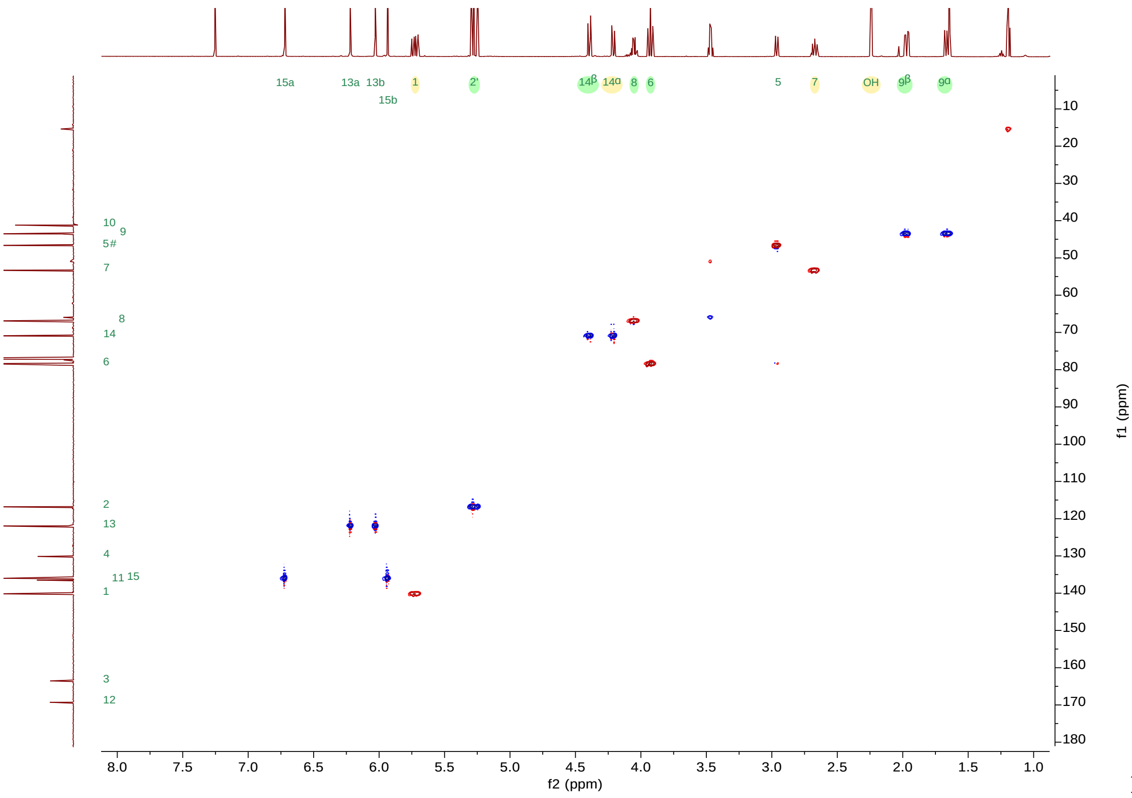

Figure S12: $^{1}H/^{13}C$- HSQC spectrum of compound **2** ($CDCl_3$, 600MHz)

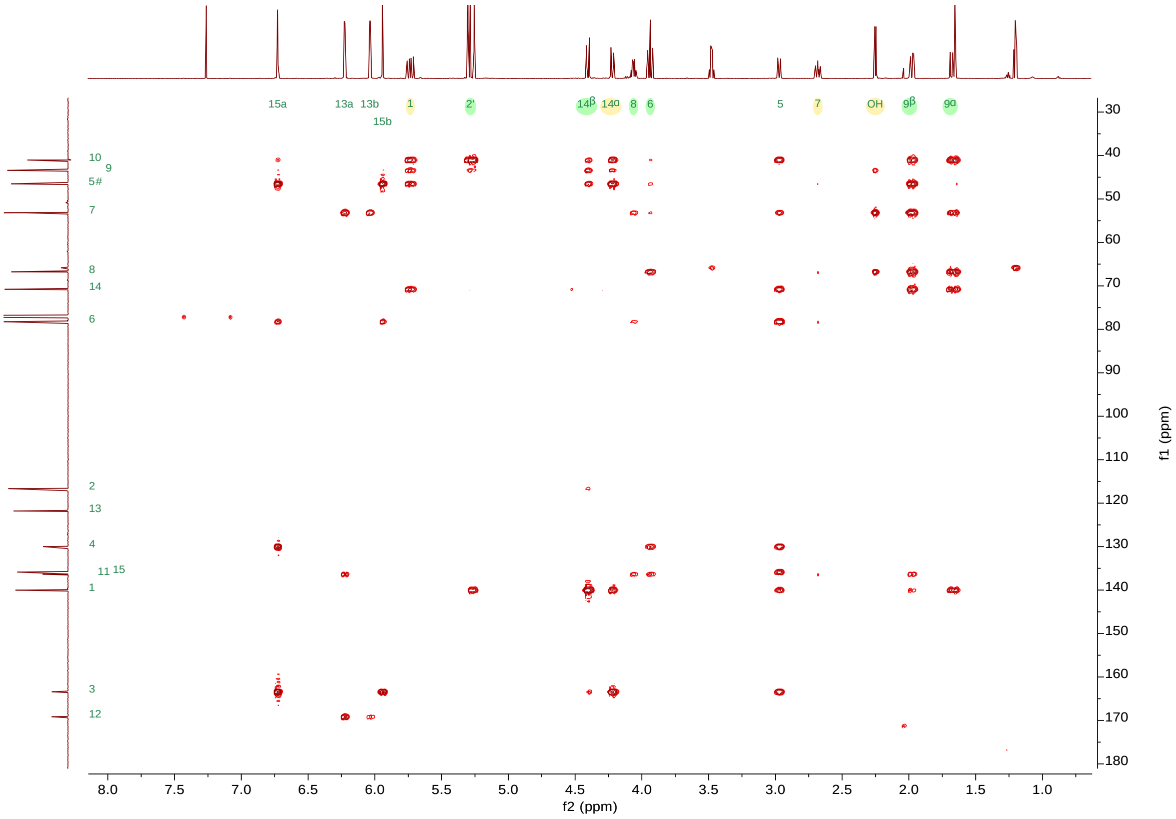

Figure S13: $^{1}H/^{13}C$- HMBC spectrum of compound **2** ($CDCl_3$, 600MHz)

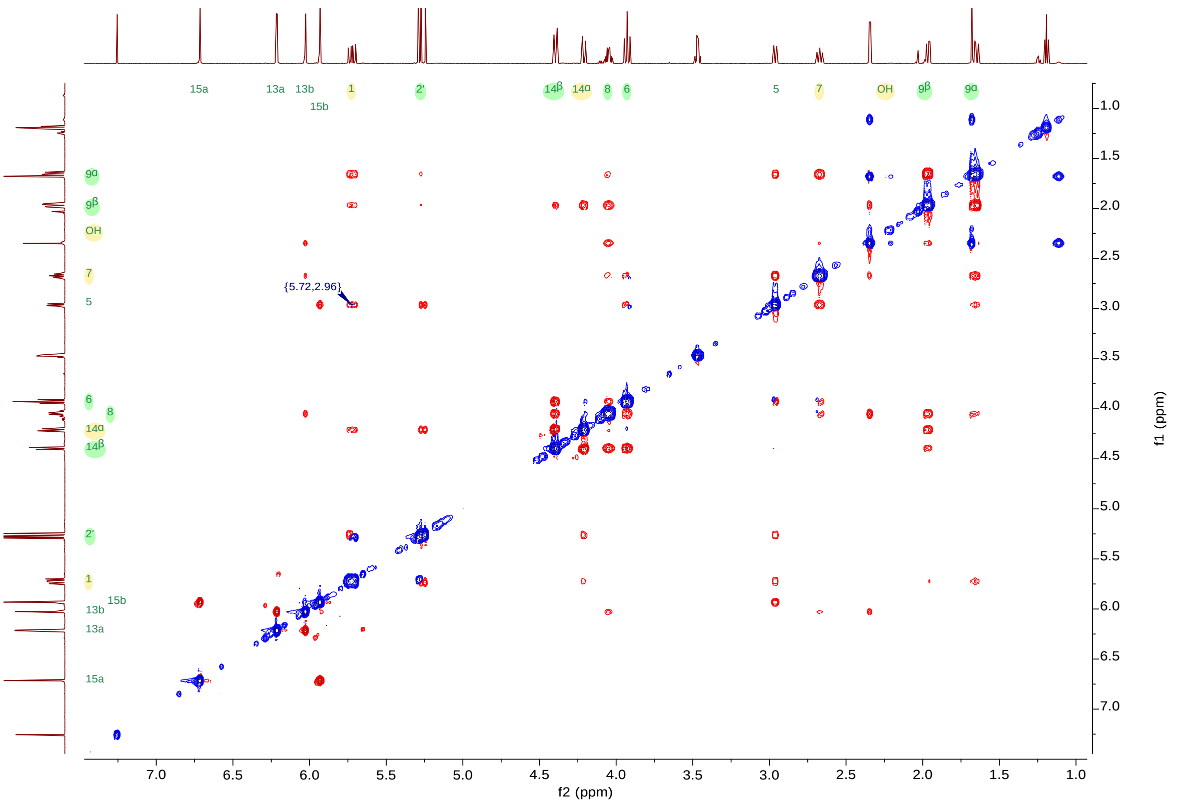

Figure S14: $^1H/^1H$- NOESY spectrum of compound **2** ($CDCl_3$, 600MHz)

Compound 3

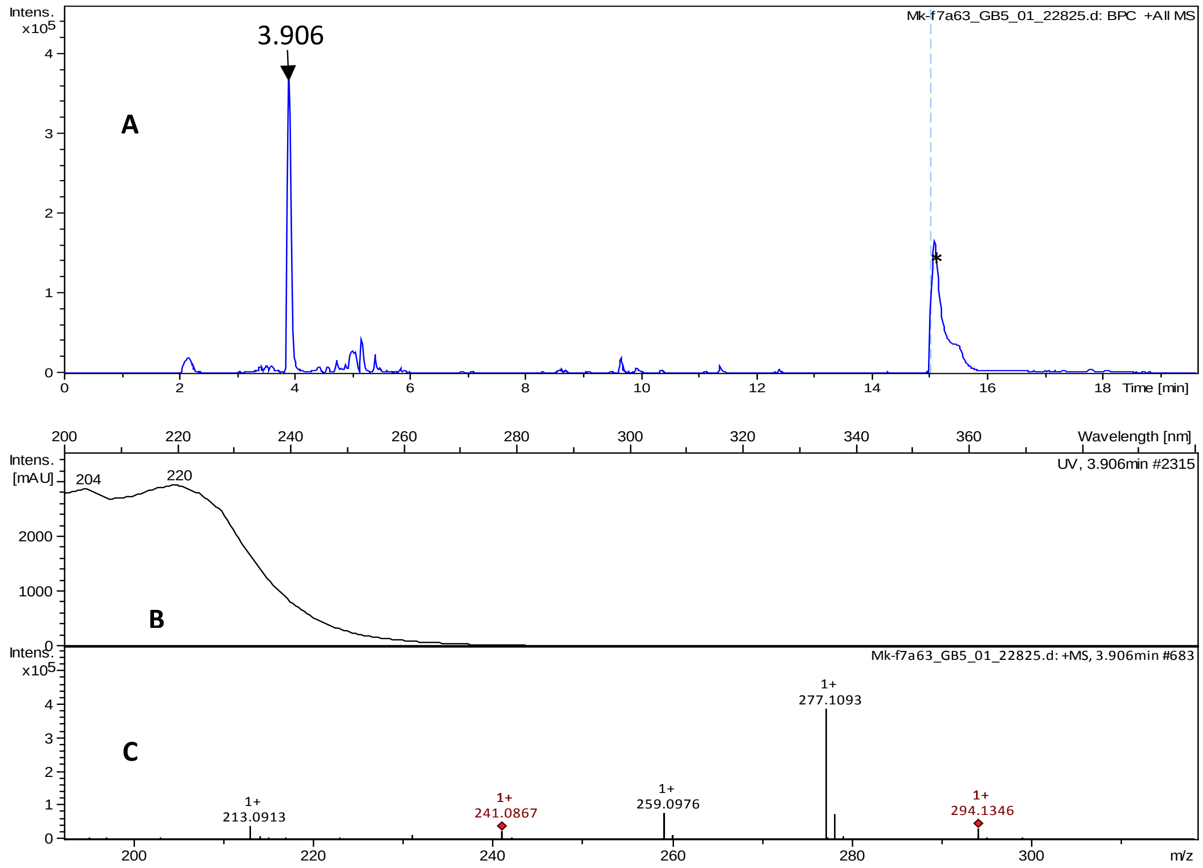

Figure S15: +ESI-QqTOF-MS chromatogram (**A**); UV spectrum (**B**) and +ESI-MS spectrum (**C**) of compound **3**, $[M + H]^+$: 277.1093 ($C_{15}H_{17}O_5$). Internal calibrant sodium formate: 15.106 min

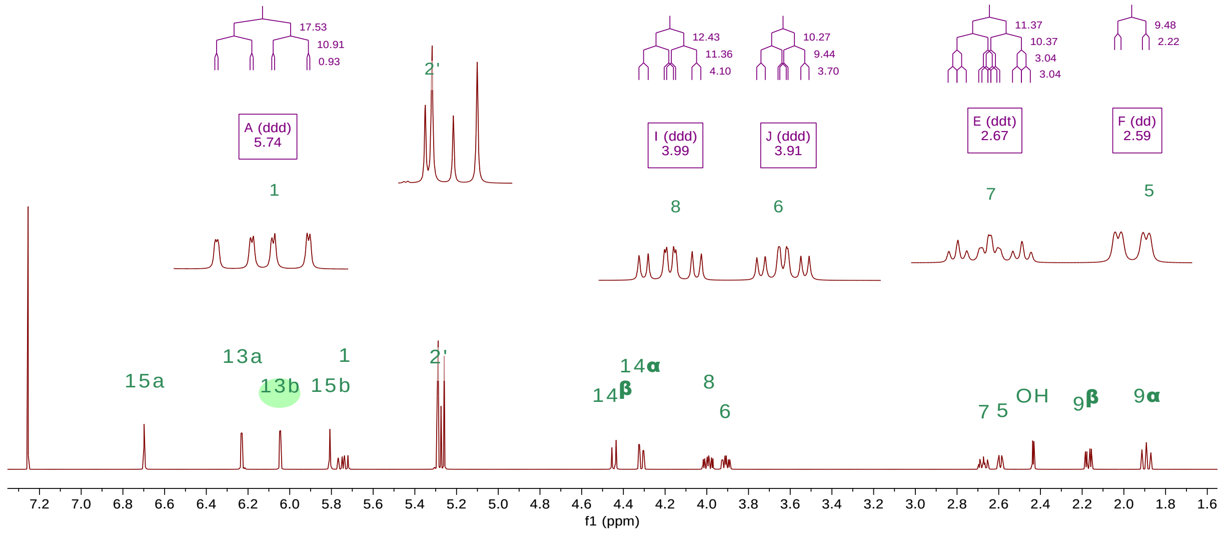

Figure S16: ^{1}H-NMR spectrum of compound **3** ($CDCl_3$, 600 MHz)

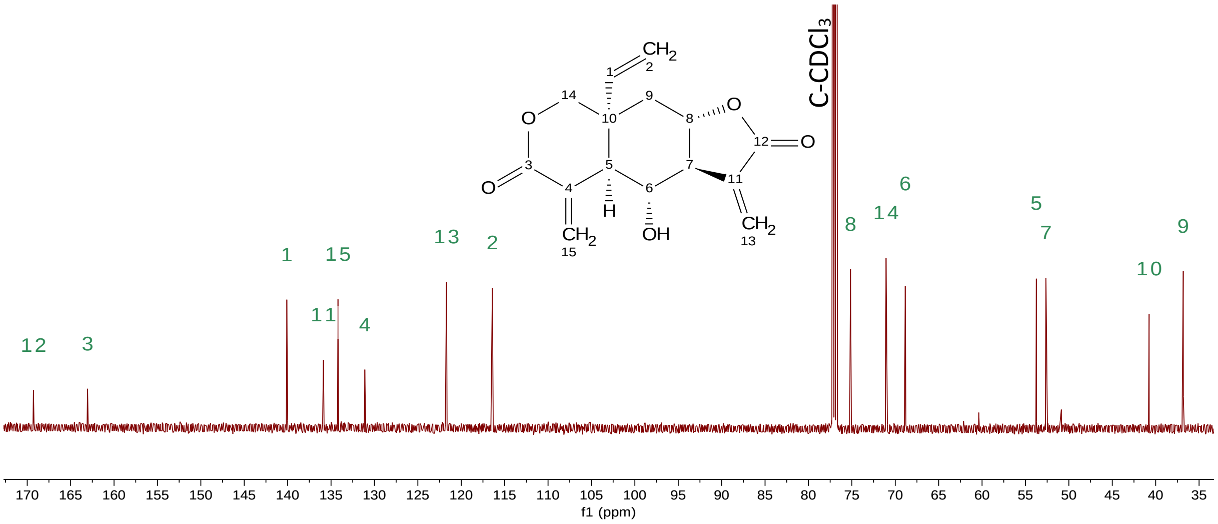

Figure S17: ^{13}C-NMR spectrum of compound 3 ($CDCl_3$, 600 MHz)

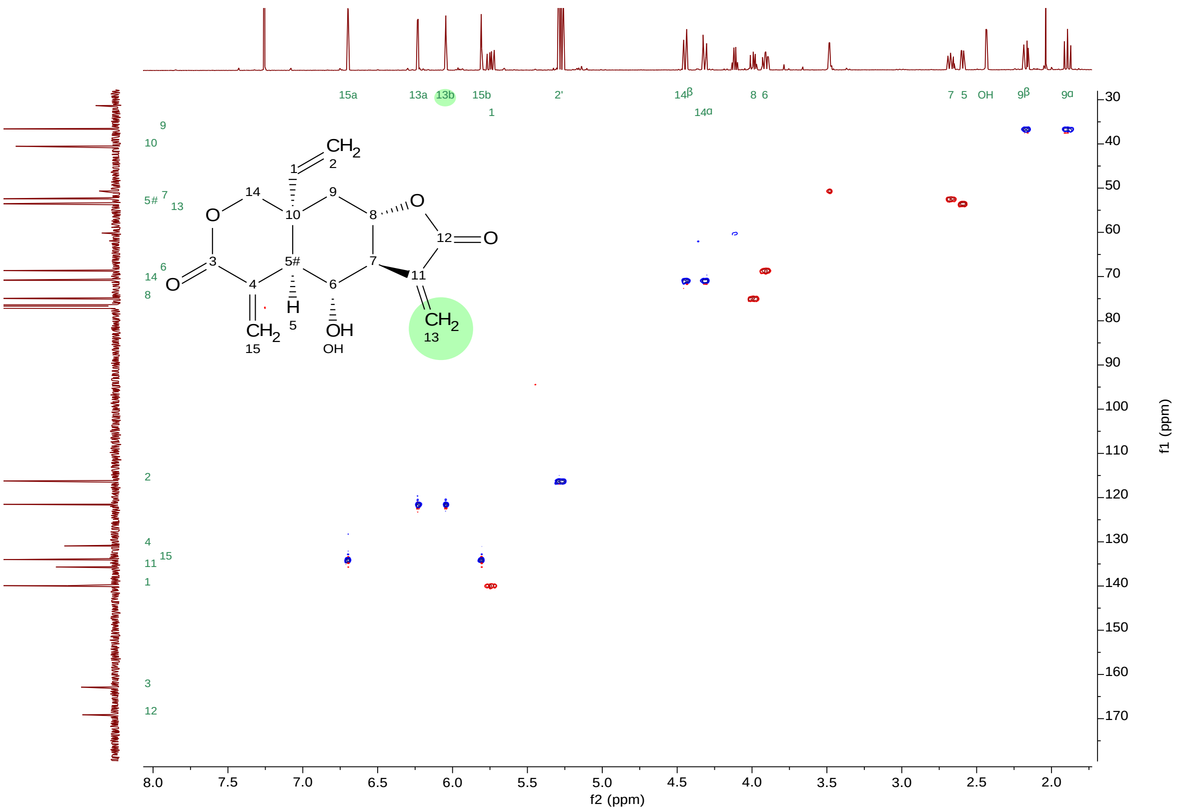

Figure S18: $^1H/^{13}C$- HSQC spectrum for compound **3** ($CDCl_3$, 600MHz)

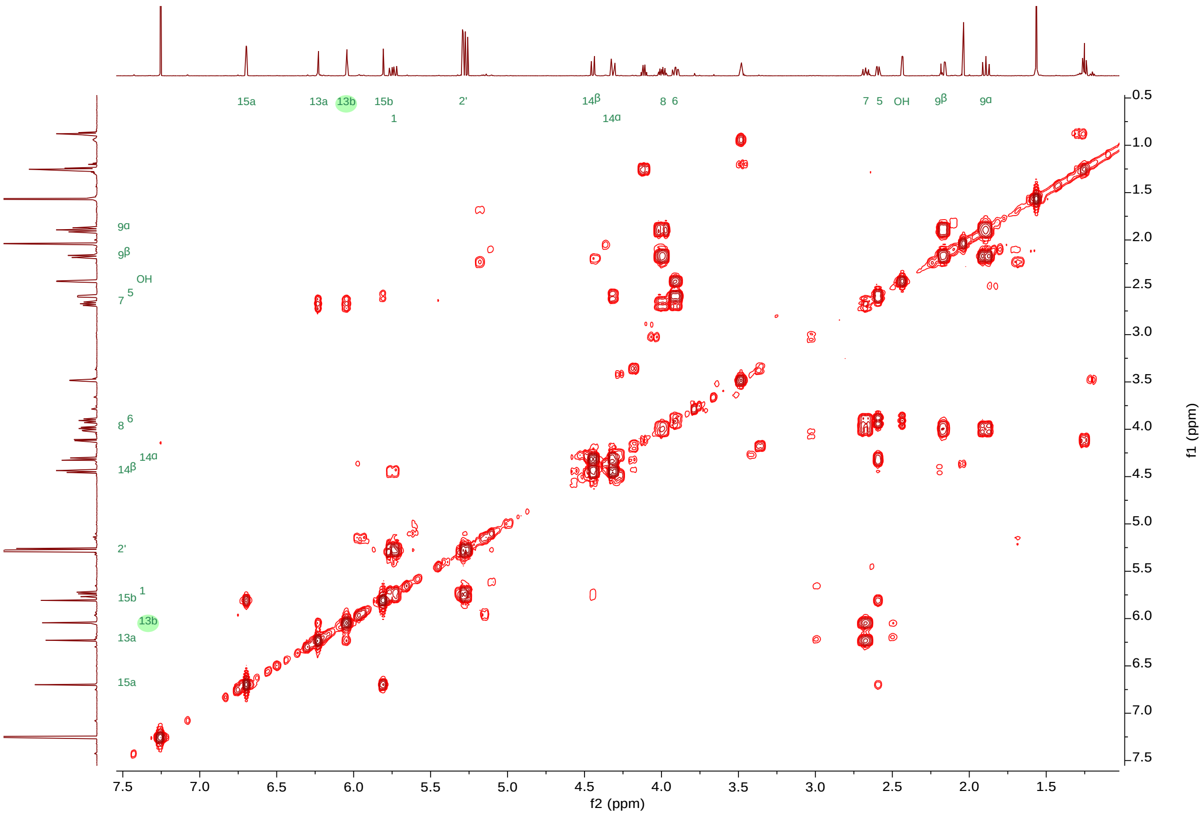

Figure S19: $^1H/^1H$- COSY spectrum for compound **3** ($CDCl_3$, 600MHz)

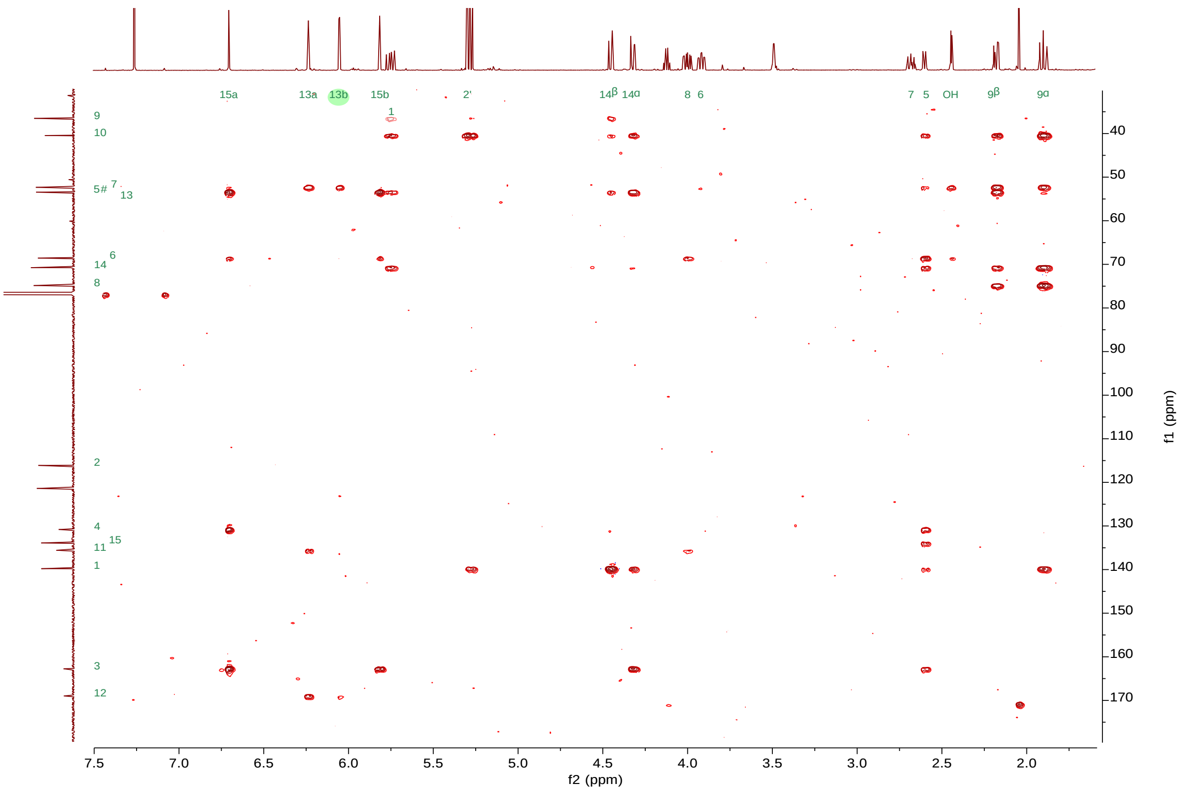

Figure S20: $^{1}H/^{13}C$- HMBC spectrum for compound **3** ($CDCl_3$, 600MHz)

Compound 4

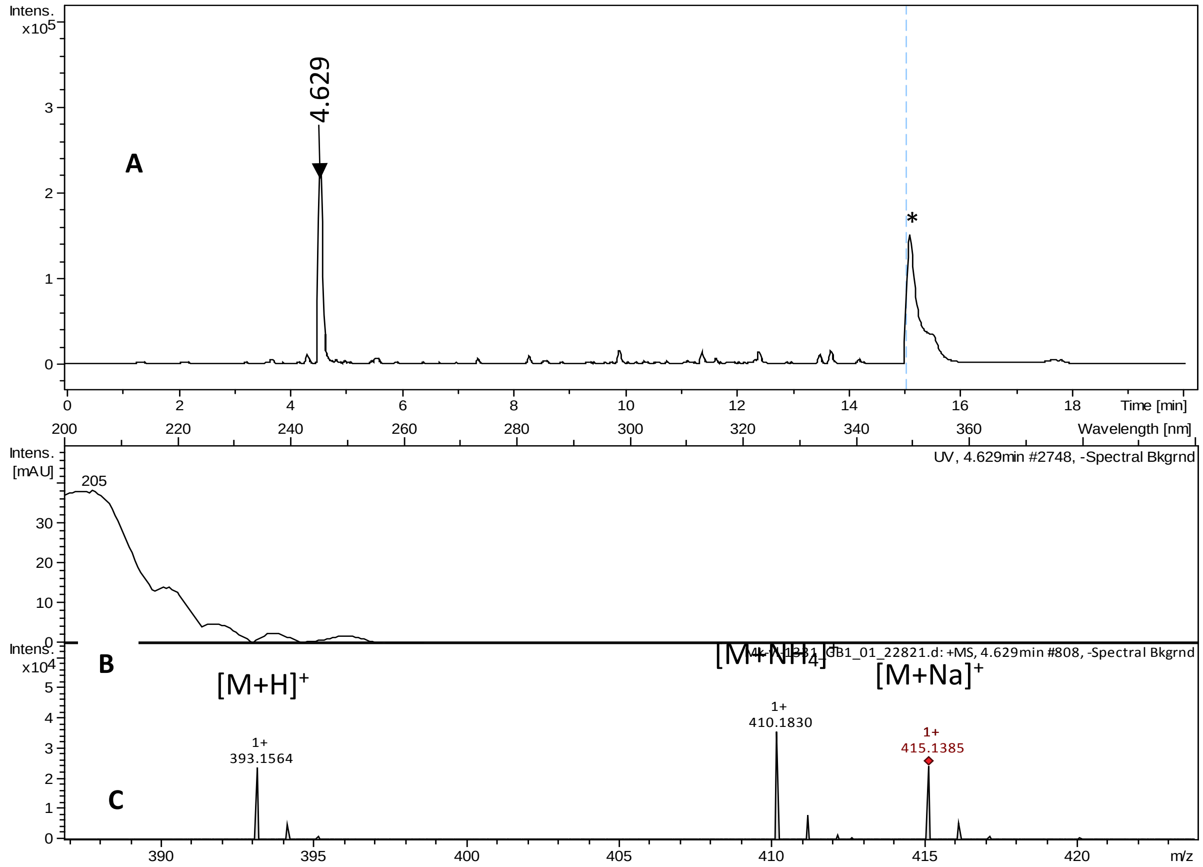

Figure S21: +ESI-QqTOF-MS chromatogram (**A**); UV spectrum (**B**) and +ESI-MS spectrum (**C**) of compound **4**, [M + H]$^+$: 393.1564($C_{20}H_{25}O_8$). Internal calibrant sodium formate: 15.106 min

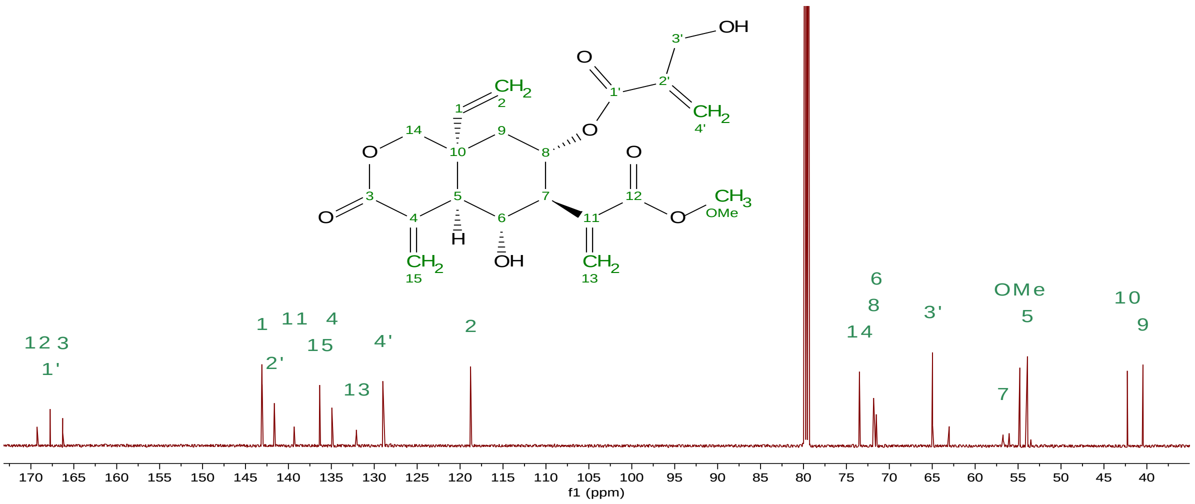

Figure S22: ^{13}C-NMR spectrum of compound **4** ($CDCl_3$, 600 MHz)

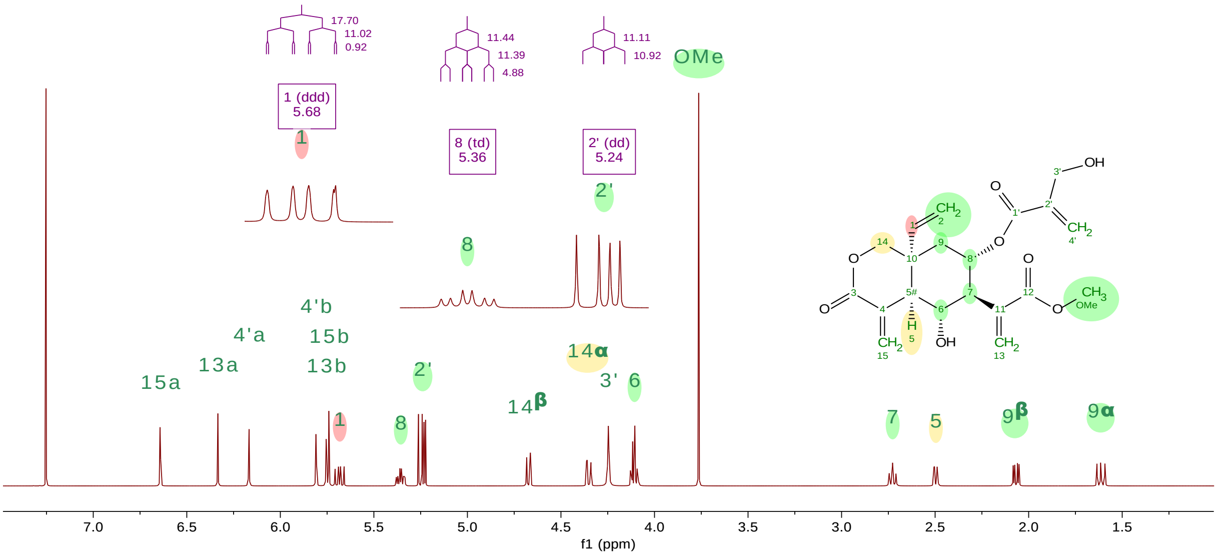

Figure S23: ^{1}H-NMR spectrum of compound **4** ($CDCl_3$, 600 MHz)

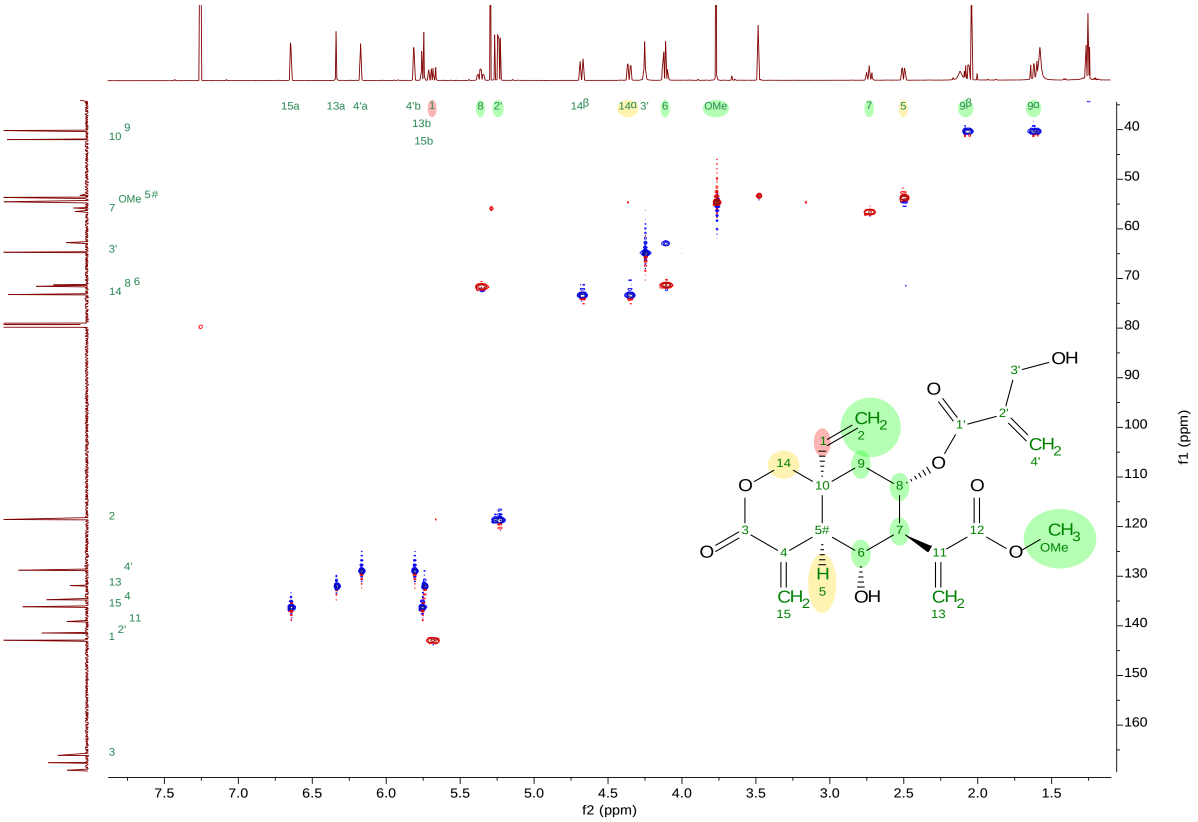

Figure S24: $^{1}H/^{13}C$- HSQC spectrum for compound **4** ($CDCl_3$, 600MHz)

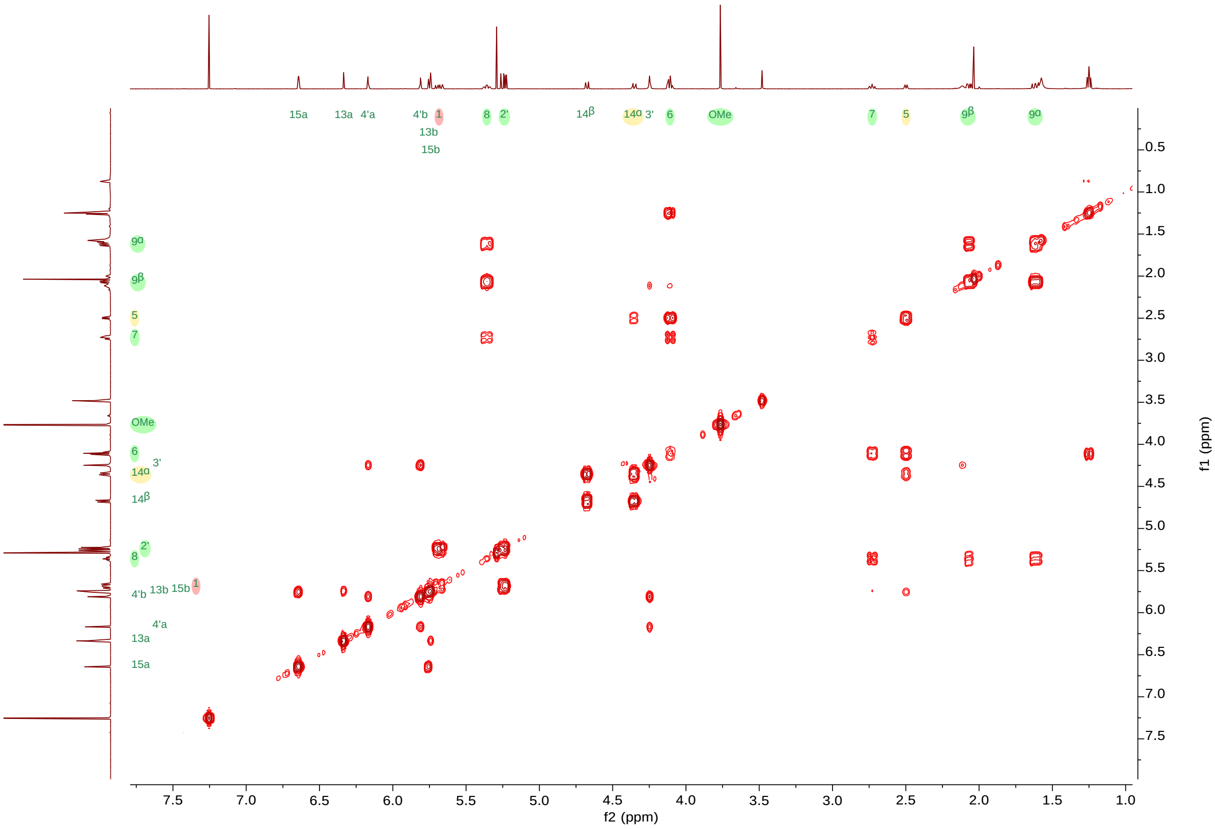

Figure S25: $^{1}H/^{1}H$- COSY spectrum for compound **4** ($CDCl_3$, 600MHz)

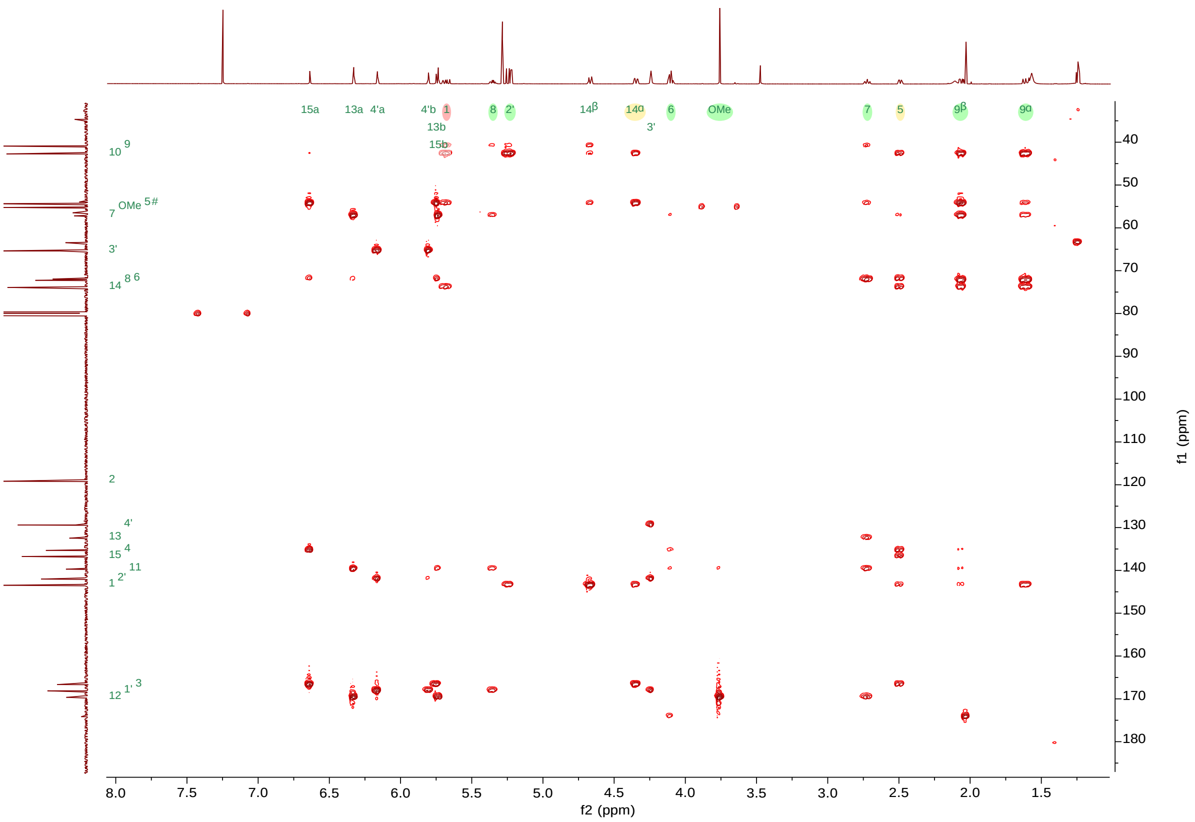

Figure S26: $^{1}H/^{13}C$- HMBC spectrum for compound **4** ($CDCl_3$, 600MHz)

Compounds **5**+**6**

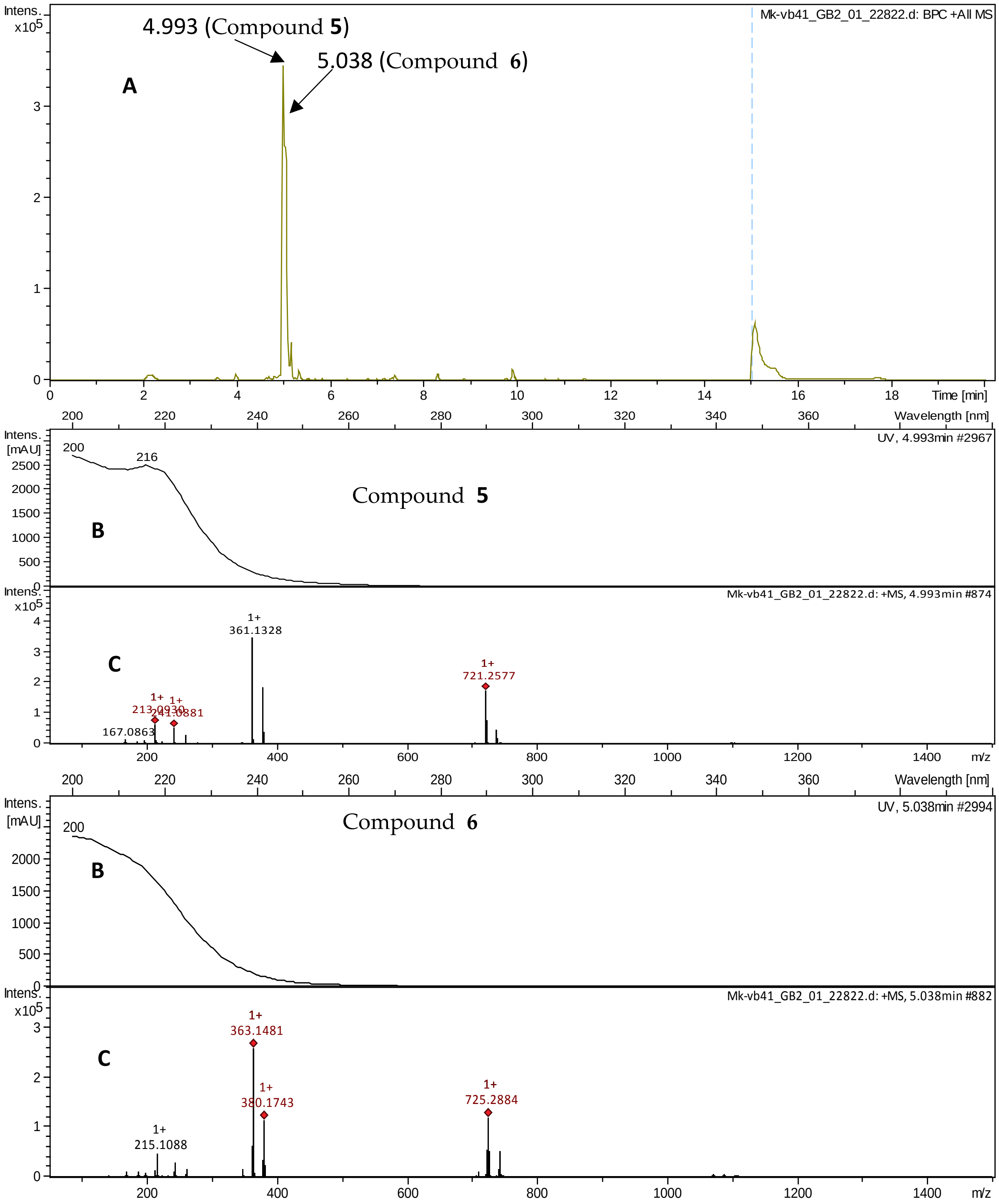

Figure S27: +ESI-QqTOF-MS chromatogram (**A**); UV spectra (**B**) and +ESI-MS spectra (**C**) of compounds **5** and **6**, $[M + H]^+$: 361.1328($C_{19}H_{21}O_7$) and $[M + H]^+$: 363.1481($C_{19}H_{23}O_7$) for Compound **5** and **6** respectively. Internal calibrant sodium formate: 15.106 min

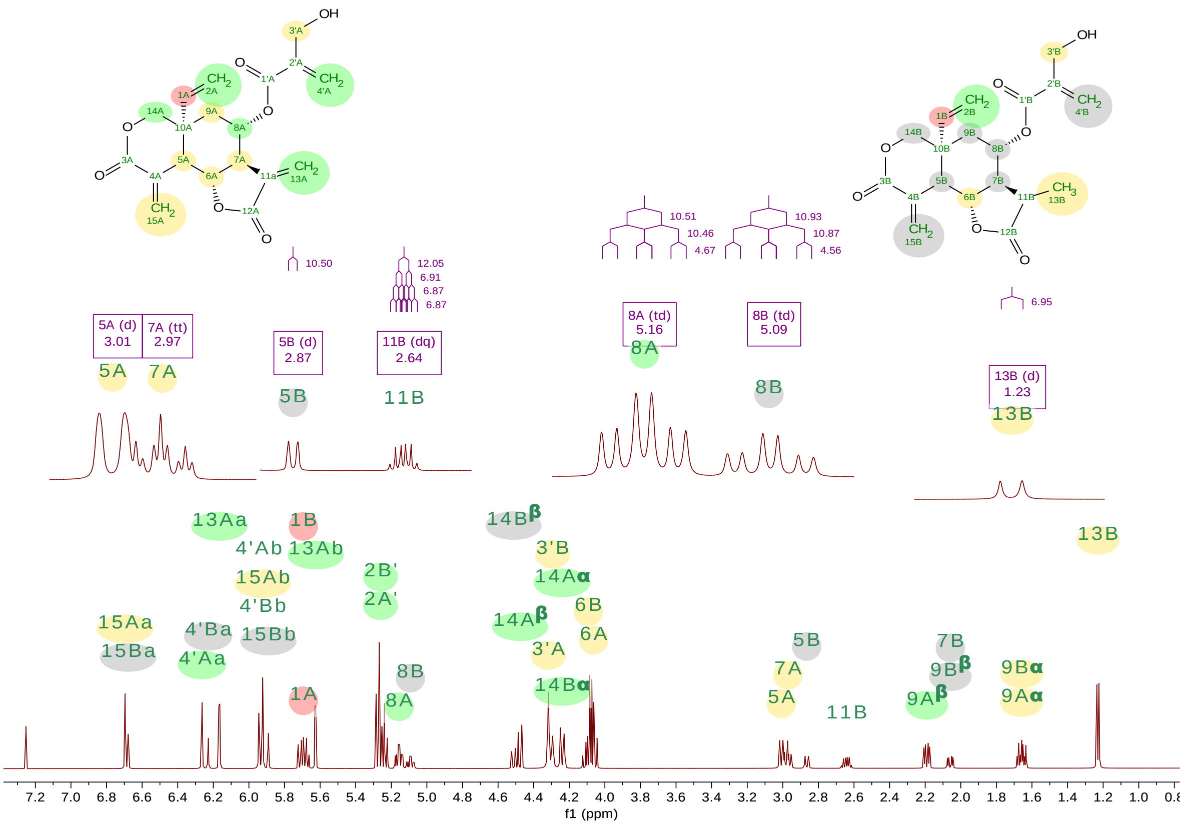

Figure S28: ^{1}H-NMR spectrum of compounds **5** (**A**) and **6** **(B)** ($CDCl_3$, 600 MHz)

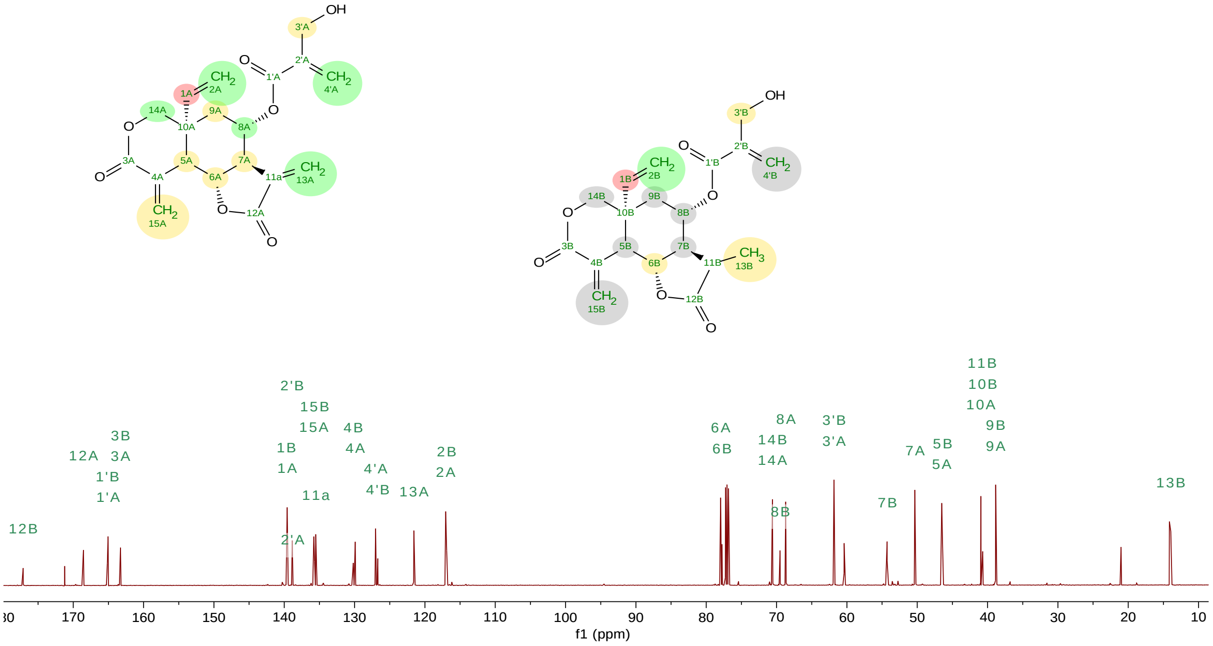

Figure S29: ^{13}C-NMR spectrum of compounds **5** (A) and **6** (B) ($CDCl_3$, 600 MHz)

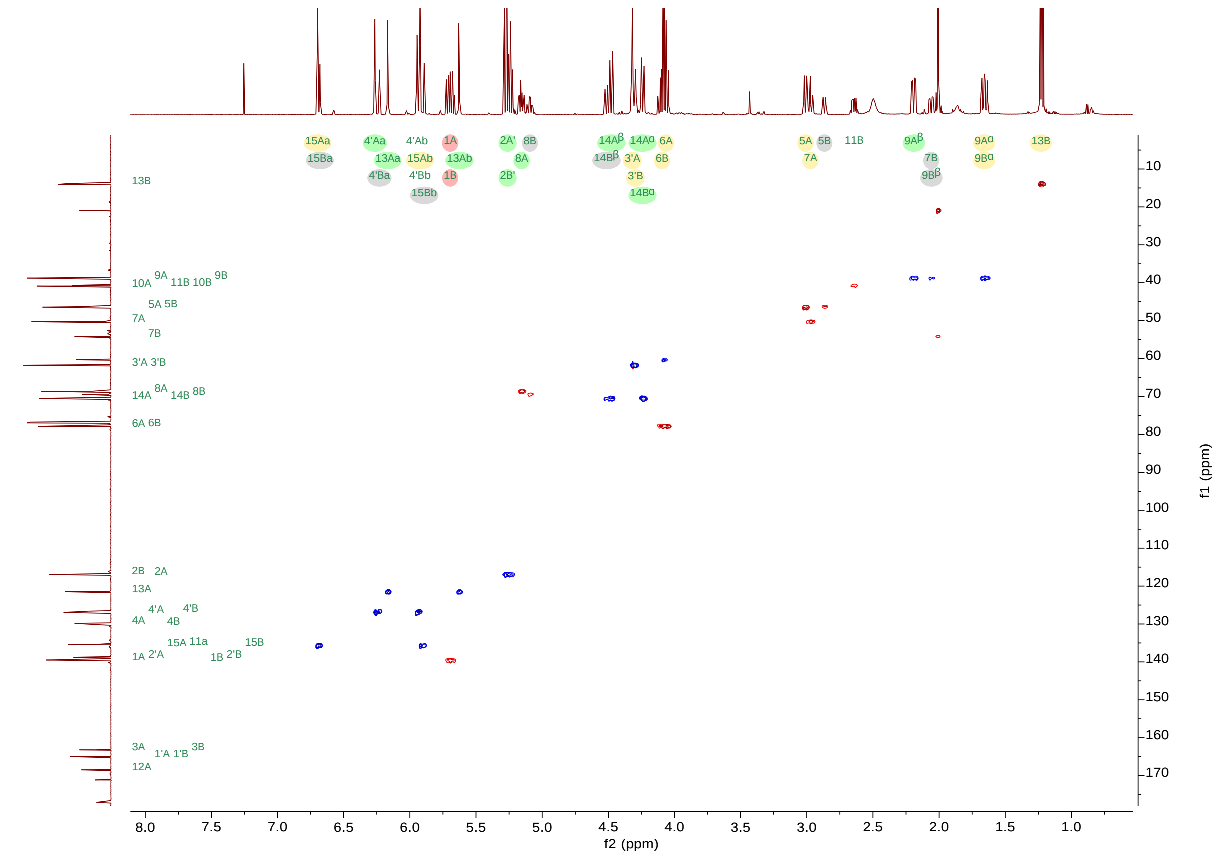

Figure S30: $^{1}H/^{13}C$- HSQC spectrum for compounds **5** (A) and **6** (B) ($CDCl_3$, 600 MHz)

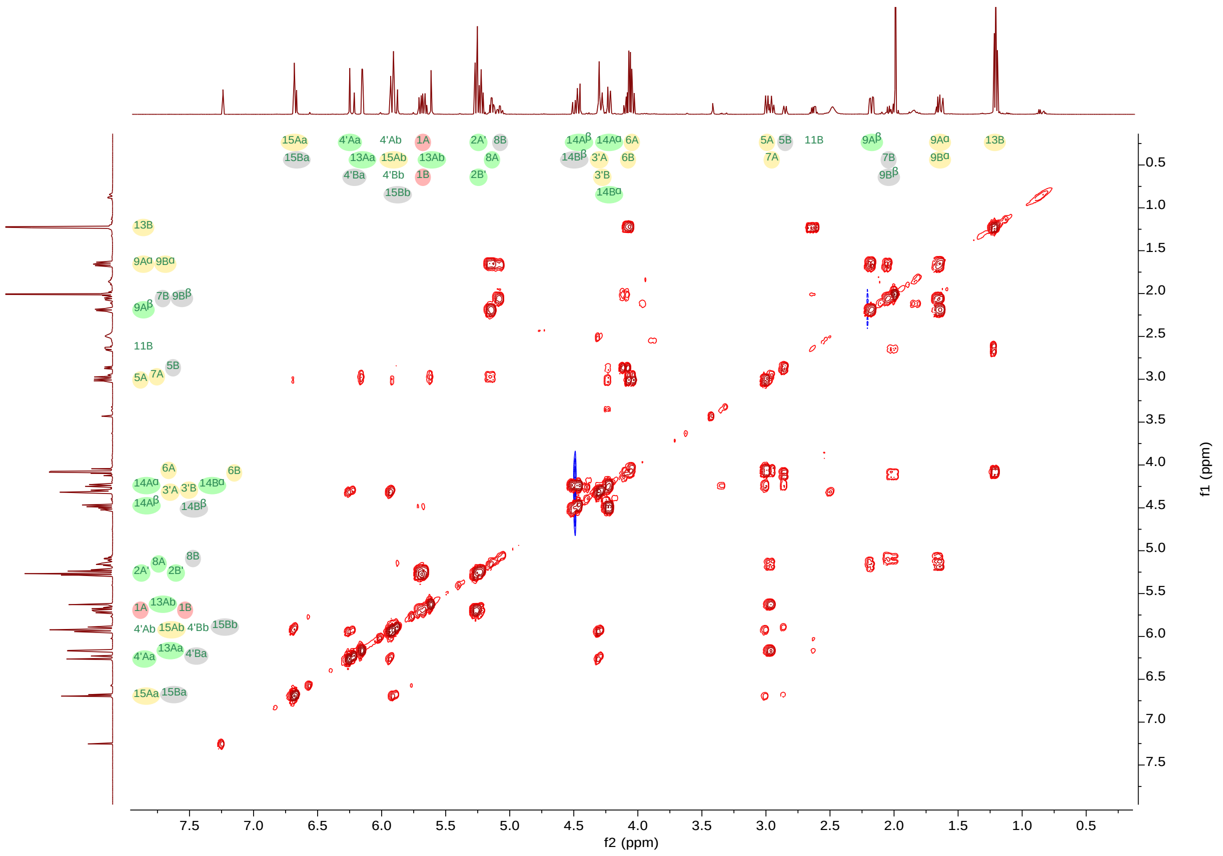

Figure S31: $^{1}H/^{1}H$- COSY spectrum for compounds **5** (A) and **6** (B) ($CDCl_3$, 600MHz)

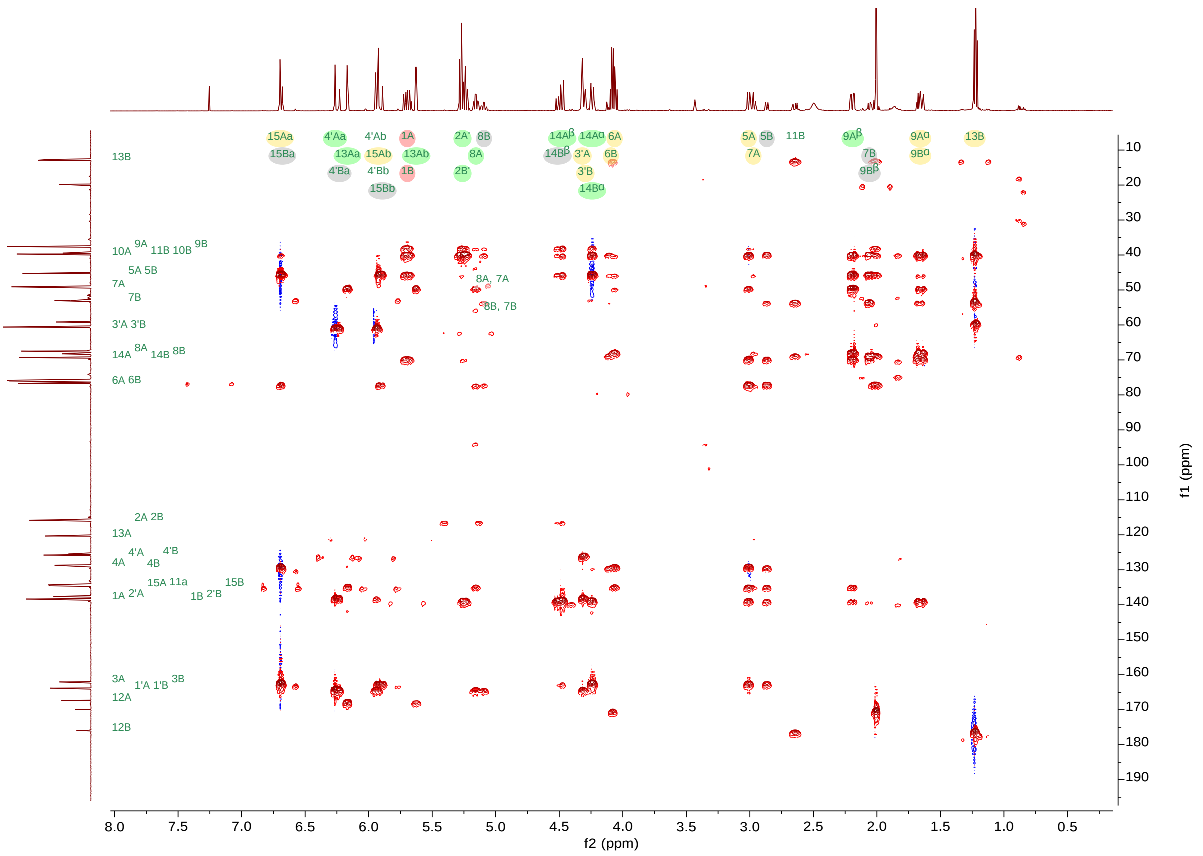

Figure S32: $^1H/^{13}C$- HMBC spectrum for compounds **5** (A) and **6** (B) ($CDCl_3$, 600MHz)
